# Inflammasome-mediated cell death pathways in female reproductive disorders: from apoptosis, pyroptosis, and necroptosis to PANoptosis

**DOI:** 10.1186/s13048-026-02221-8

**Published:** 2026-07-29

**Authors:** Amirhossein Mardi, Hamid Ahmadi, Samaneh Abdolmohammadi-Vahid, Pouya Zahmatkesh, Golaleh Savari, Mohammad Sadegh Soltani-Zangbar, Leili Aghebati-Maleki

**Affiliations:** 1 Immunology Research Center, Tabriz University of Medical Sciences, Tabriz, Iran; 2 Department of Immunology, School of Medicine, Tabriz University of Medical Sciences, Tabriz, Iran; 3 Department of Medical Biology, Medical School, University of Pécs, Pécs, 7624 Hungary; 4 János Szentágothai Research Centre, University of Pécs, Pécs, 7624 Hungary

**Keywords:** PANoptosis, Pyroptosis, Necroptosis, Apoptosis, Reproductive diseases

## Abstract

Female reproductive disorders, including endometriosis, polycystic ovary syndrome (PCOS), preeclampsia (PE), recurrent pregnancy loss (RPL), premature ovarian insufficiency (POI), and implantation-related complications, are frequently associated with chronic inflammation, immune dysregulation, and impaired tissue homeostasis. Increasing evidence suggests that inflammasomes play central roles in regulating inflammatory responses and multiple forms of programmed cell death, including apoptosis, pyroptosis, necroptosis, and the recently described integrated cell death pathway known as PANoptosis. Inflammasome activation promotes the maturation of pro-inflammatory cytokines such as interleukin (IL)-1β and IL-18 and contributes to cellular and tissue injury through downstream cell death signaling pathways. Here, we review the existing knowledge on the molecular mechanisms linking inflammasome signaling to the regulated cell death pathways in female reproductive disorders. The role of apoptosis, pyroptosis and necroptosis in the pathogenesis of endometriosis, PCOS, PE, RPL, POI and other pregnancy related complications and the role of NLRP3 and other inflammasome complexes in disease progression are discussed. Recent studies suggest that crosstalk between these cell death pathways can lead to inflammatory pathology via PANoptosis-related mechanisms, although direct evidence for PANoptosis in reproductive diseases is still lacking. We also review current and potential therapeutic approaches targeting inflammasomes and associated cell death pathways such as NLRP3 inhibitors, IL-1β blockade and regulators of pyroptotic signaling. Together, the available evidence suggest that inflammasome-associated cell death pathways are important contributors to reproductive disease pathogenesis and may offer new diagnostic and therapeutic opportunities. Further mechanistic and clinical studies are needed to elucidate the role of PANoptosis and determine whether targeting key inflammasome and PANoptosis-related regulators can improve reproductive outcomes.

**Graphical abstract:**

**Figure:**
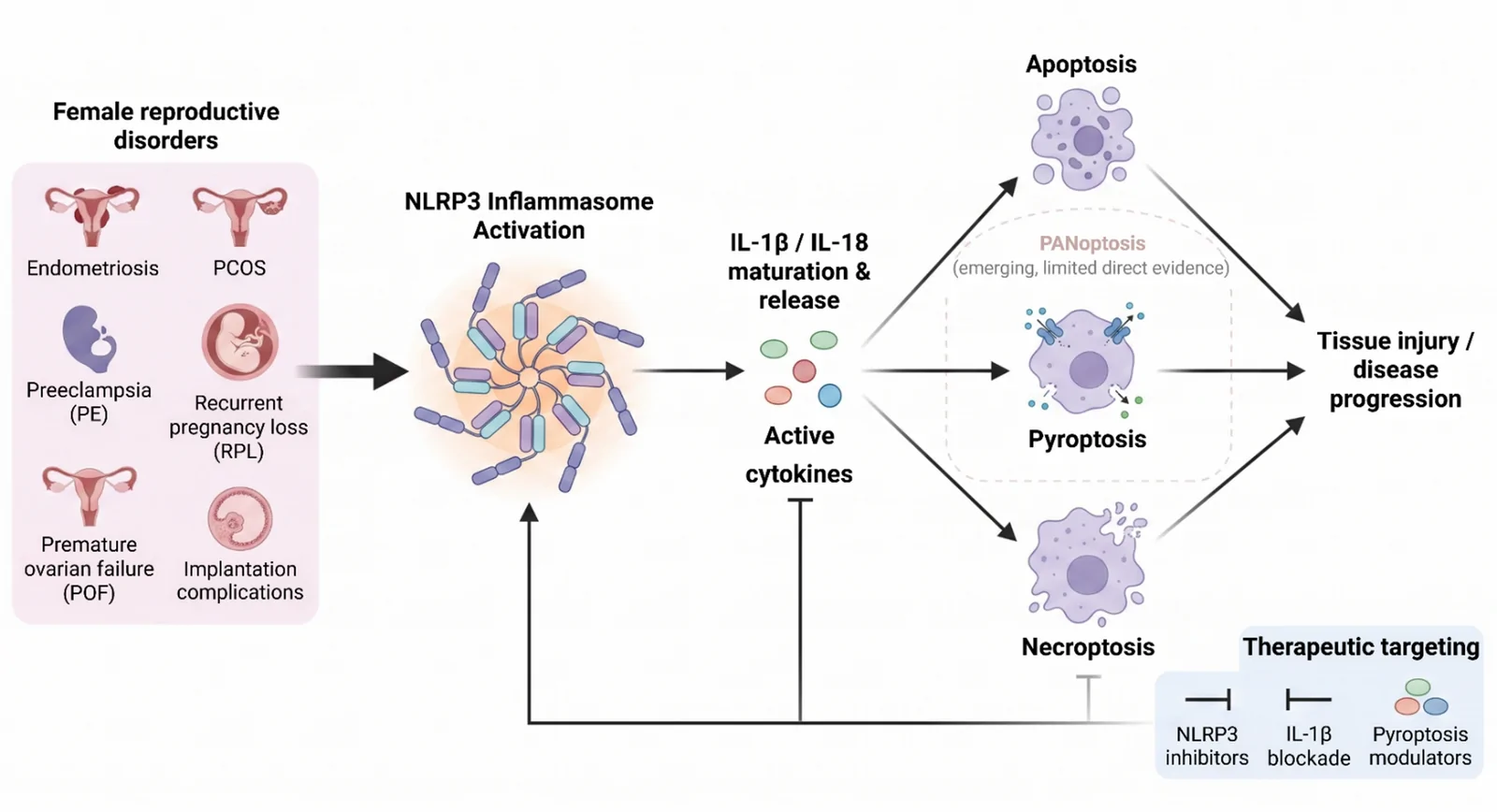

## Introduction

Programmed cell death (PCD) is a regulated, autonomous process that maintains intracellular homeostasis through the actions of different biomolecules [1, 2]. By categorizing cell death according to morphological traits, triggering mechanisms, and cellular context, researchers have lately discovered novel types of PCD, such as necrosis, autophagy, pyroptosis, cuproptosis, apoptosis, and necrosis [3]. Prior research mainly focused on the mechanics and roles of distinct forms of PCD. PCD is essential for controlling pathological and physiological processes, such as immunological responses, inflammation, and tissue formation [4]. However, new information suggests that PCDs create a complicated network of interactions rather than operating independently [4, 5]. PANoptosis is a distinctive process of programmed cell death associated with innate immune-inflammatory responses, as revealed by Malireddi et al. in 2019 [6]. PANoptosis is regulated by the PANoptosome complex, which entails collaboration and interactions between apoptosis, necrosis, and pyroptosis. However, no single type of PCD can be held responsible for the full biological effects of PANoptosis [7].

PANoptosis combines essential elements of necrosis, pyroptosis, and apoptosis. Pattern recognition receptors (PRRs) start signaling cascades that regulate PANoptosis by identifying damage-associated molecular patterns (DAMPs) and pathogen-associated molecular patterns (PAMPs) [8]. By starting the creation of the PANoptosome complex and triggering downstream execution molecules, including caspase-3/7, gasdermin E (GSDME), gasdermin D (GSDMD), and mixed lineage kinase domain-including (MLKL), this results in membrane perforation and cell lysis [9]. PANoptosis addresses a gap in understanding the interactions among molecular components of different PCD pathways. It emphasizes the coordination and communication between necrosis, apoptosis, and pyroptosis in reaction to pathogens and innate immune stimuli [10].

Chronic inflammation, a dysregulated immune response, and improper cell death in reproductive organs are often characteristics of reproductive health problems, such as infertility, spontaneous miscarriage, endometriosis, or recurrent pregnancy loss [11]. The discovery of PANoptosis, a programmed cell death process that synchronizes pyroptosis, apoptosis, and necroptosis processes, as a potentially important contributor to reproductive disorder disease pathways, defines this new area of innate immunity. Increased cytokines, immune cell infiltration, or oxidative stress are characteristics of inflammatory microenvironments in reproductive tissues (such as the uterus, fallopian tubes, and ovarian tissues), including endometriosis [12–14]. These processes may prime cells for PANoptotic activation. Local inflammation and scarring caused by endometriosis hinder ovulation, result in failed embryos, and cause spontaneous loss of the embryo [15, 16]. Although simultaneous activation of apoptosis-, pyroptosis-, and necroptosis-related signaling has been reported in several reproductive disorders, direct evidence demonstrating canonical PANoptosome assembly or bona fide PANoptosis in reproductive tissues remains scarce. Therefore, throughout this review, PANoptosis is discussed as an emerging mechanistic framework that may integrate these regulated cell death pathways rather than as a definitively established mechanism in female reproductive diseases.

Although substantial progress has been made in understanding the roles of inflammasomes and individual regulated cell death pathways in reproductive biology, the complex interactions among apoptosis, pyroptosis, necroptosis, and the emerging concept of PANoptosis remain incompletely understood. Growing evidence suggests that dysregulated inflammasome signaling contributes to chronic inflammation, immune imbalance, and tissue dysfunction in a wide range of reproductive disorders; however, the underlying molecular mechanisms and their therapeutic implications have not been comprehensively evaluated. Therefore, this review aims to summarize current knowledge regarding inflammasome-mediated cell death pathways in female reproductive disorders, with particular emphasis on the roles of apoptosis, pyroptosis, necroptosis, and PANoptosis in disease pathogenesis. Furthermore, we discuss the crosstalk among these pathways, evaluate emerging evidence supporting PANoptosis-related mechanisms, and highlight current and potential therapeutic strategies targeting inflammasome-associated signaling networks in reproductive diseases.

## Inflammasomes: structure and activation

### NLRP3 inflammasome

Three common components may be used to distinguish and characterize the formation of the NLRP3 inflammasome as a signaling cascade: sensor, adaptor, and effector [17]. NLRP3, ASC (apoptosis-associated speck-like protein containing a caspase recruitment domain), and caspase-1, respectively, are responsible for these functions. An amino (N)-terminal pyrin domain (PYD), a carboxy (C)-terminal LRR, and a central ATPase domain termed NACHT that contains NBD make up the sensor NLRP3 [18]. To control downstream signaling, PYDs often engage in homotypic interactions. Protein-protein interactions are mostly mediated by LRR domains, and NLRP3’s LRR has also been linked to NLRP3 stability [18]. Once activated, NACHT domains, which have the capacity to bind nucleotides and hydrolyze ATP, may undergo ATP-dependent self-oligomerization, which produces self-PYD contacts that further attract ASC, the adaptor [18]. A large catalytic subunit known as p20, a minor catalytic subunit known as p10 at the C-terminal, and an N-terminal CARD make up caspase-1 [19]. Similar nucleated filament development occurs between the caspase-1 CARDs and the clustered ASC CARDs. The p20 and p10 catalytic subunits of caspase-1 undergo proximity-induced dimerization and self-cleavage at the p20–p10 linker as a result of these filaments, rendering caspase-1 completely proteolytically active [20]. When caspase-1 is active, it may cleave and activate GSDMD, which causes pyroptosis, and transform pro-cytokines in the IL-1 family into mature proinflammatory cytokines, which are crucial for controlling immunological responses [21].

### AIM2 inflammasome

The AIM2 receptor is a member of the hematopoietic interferon-inducible nuclear (HIN) domain (HIN) and pyrin (pyrin + HIN) family. It is composed of a C-terminal HIN domain that recognizes cytosolic DNA and an N-terminal PYD that recruits the adaptor protein apoptosis-associated speck-like protein containing a CARD (caspase recruitment domain) (ASC) [22]. Hematopoietic cells constitutively express AIM2, which is directly triggered by dsDNA produced by cells infected with *Listeria*, *Francisella*, or viruses (such as *Vaccinia* and *Cytomegalovirus*), as well as by endogenous DNA released following cell injury [23]. Active IL-1β and IL-18 are released when AIM2 recognizes DNA/DNA-RNA hybrids and assembles the inflammasome complex, which is characterized by caspase-1 (canonical route) or caspase-4/−8 (non-canonical pathway) [24]. AIM2 does not require the two-signal paradigm, unlike other sentinel receptors (NLRs, NOD-like receptors), hence its expression is independent of the priming signal, unlike NLRP3 [25]. However, it should be mentioned that the cGAS (cyclic GMP-AMP synthase)-STING (stimulator of interferon genes) signaling pathway is another cytoplasmic dsDNA sensor that has been identified as being in charge of the type I interferon (IFN) response [26].

### NLRP1 inflammasome

NLRP1 is structurally composed of a leucine-rich repeat (LRR) region and a central nucleotide-binding domain (NBD) [27]. Most, if not all, of the mammalian species whose genomes have been sequenced, including primates, rodents, ungulates, and even marsupials, have NLRP1-like genes [28]. Like other NLRs, NLRP1 has a short LRR and a central NBD. There are two identified effector-recruitment domains in NLRP1 [28]. These two domains are the CARD and PYD. These two effector domains are structurally connected to one another since they belong to the death domain superfamily. The PYD and CARD domains are thought to facilitate homotypic interactions with other downstream proteins that include PYD/CARD, such as ASC and caspase-1 [29]. The first NLR that was shown to create a cytosolic inflammasome complex that could attract and activate caspase-1 was NLRP1 [28]. In order to identify NLRP1’s capacity to create inflammasomes, early research relied on spontaneous activation of the protein seen in cell lysates. Nevertheless, only a portion of the exact mechanisms and physiological activators of NLRP1 activation have been identified [28].

## Regulated cell death pathways

### Apoptosis

A tight intracellular arrangement of organelles, nuclear division, cell shrinkage, and the development of an apoptotic body in the cytoplasm are the hallmarks of apoptosis, a type of controlled cell death [30]. Apoptosis primarily occurs through two pathways: the intrinsic and extrinsic pathways. DNA damage, Endoplasmic reticulum stress, and other endogenous apoptotic signals trigger the inherent, or mitochondrial, apoptotic pathway. Apoptosis is triggered by changes in mitochondrial structure and function in this pathway [30, 31]. BCL-2 family members that promote apoptosis (BAK, BAX, Bid, etc.) regulate the intrinsic pathway by encouraging the release of cytochrome c [32, 33]. Pro-caspase-9 is subsequently activated by the apoptosome, a complex that cytochrome c forms in the cytoplasm with Apaf-1 and dATP [34].

**Fig. 1:**
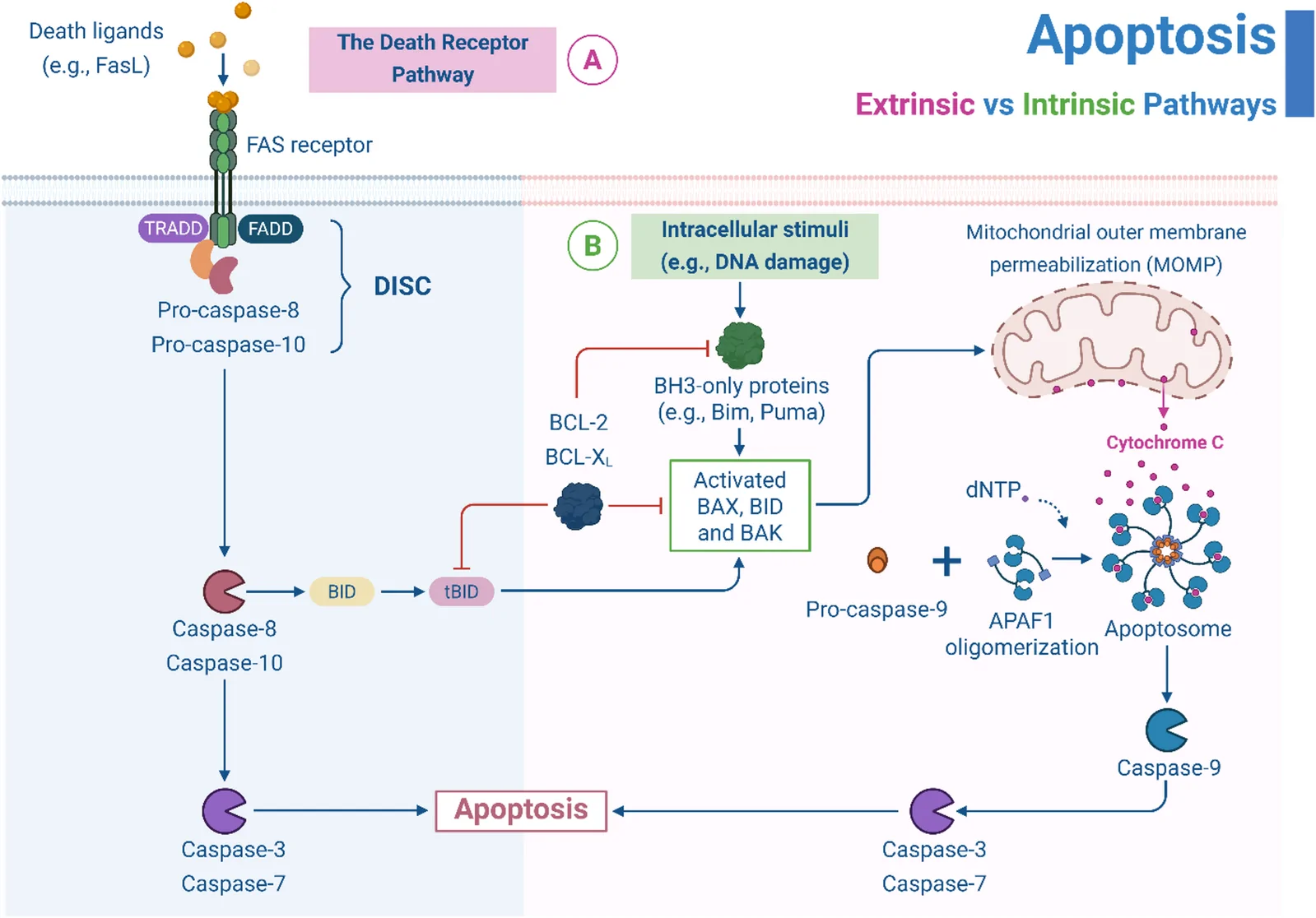
Diagrammatic representation of the intrinsic and extrinsic apoptotic pathways. This illustration shows the two key apoptotic signaling pathways: (**A**) death receptor-mediated extrinsic pathway and (**B**) mitochondrion-mediated intrinsic pathway. The extrinsic pathway (**A**) is the binding of extracellular death ligands, e.g., FasL, to the FAS receptor. This is followed by adaptor protein FADD and TRADD recruitment, leading to activation of the death-inducing signaling complex (DISC). This cascade initiates the activation of pro-caspase-8 and pro-caspase-10, which results in the activation of downstream effector caspases (caspase-3 and − 7) with a final result of apoptotic cell death. Activated caspase-8 also cleaves BID to its truncated form (tBID), thereby establishing a connection between the extrinsic and intrinsic pathways. The intrinsic pathway (**B**) involves the activation of BH3-only proteins, e.g., Bim and Puma, by intracellular stress signals such as DNA damage. The proteins induce the activation of mitochondrial outer membrane permeabilization (MOMP) when they interact with pro-apoptotic Bcl-2 family proteins, i.e., BAX, BAK, and BID. The release of cytochrome c from the mitochondria results in the activation of the apoptosome when it interacts with Apaf-1, procaspase-9, and dATP, which activates caspase-9. The two pathways activate executioner caspases (caspases-3 and − 7) ultimately, which induce characteristic apoptotic morphological changes and controlled cell death. This mechanism maintains tissue homeostasis and dampens inflammation, hence distinguishing apoptosis from other forms of regulated cell death

Cell-surface receptors for death and their ligands facilitate the extrinsic mechanism of apoptosis [35]. Death receptors with death domains, such as FADD and TRADD, are implicated in the extrinsic route. After identifying particular ligands, these death receptors assemble and trigger caspase-8 to create a death-inducing signaling complex (DISC). Subsequently, caspase-8 triggers the intrinsic route or executioner caspases [36]. Overall, several essential components are required to mediate apoptosis, including proteins from the Bcl-2 and caspase families. Every Bcl-2 family member plays a unique role in apoptosis. BCL-XL and BCL-2 inhibit apoptosis, while BAK and BAX promote it [37, 38]. The cleavage of apoptosomes is how caspase proteins, such as caspase-9, become active. They then hydrolyze other intracellular proteins [30]. The main morphologic features, triggers, and players of apoptosis are listed in Table 1 and illustrated in Fig. 1.

**Table 1:** The main morphologic features, triggers, and players of different forms of PCD

Programmed cell death	Morphologic features	Triggers	Main players Pathways
Apoptosis	ϖ Tight intracellular arrangement of organelles ϖ Cell shrinkage ϖ Nuclear division ϖ Presence of an apoptotic body in the cytoplasm	**For the intrinsic route**: DNA damage, Endoplasmic reticulum stress, and other endogenous apoptotic signals **For the extrinsic route**: cell membrane death receptors with death domains, like FADD and TRADD, and external ligands	**Intrinsic route (mitochondrial apoptotic pathway)**: BCL-2 family, BAK, BAX, Bid, cytochrome c, Apaf-1, pro-caspase-9, apoptosome, and dATP **Extrinsic pathway**: FADD and TRADD, Caspase-8
Pyroptosis	ϖ Pore formation ϖ Cell swelling ϖ Osmotic lysis ϖ Chromatin condensation ϖ DNA breakage ϖ Release of proinflammatory substances	**Granzyme-mediated route**: GzmB, GzmA **Canonical route**: PAMPs and DAMPs **Non-canonical route**: DAMPs and PAMPs like cytosolic LPS **Caspase-3/8-dependent route**: hypoxia, chemotherapy agents, inhibition of TAK1 (e.g., by Yersinia)	**Granzyme-mediated route**: GzmA, GzmB, caspase-3, GSDME, GSDMB, GSDMC, PD-L1, STAT3, Caspases-8, TNFα **Canonical route**: PAMPs and DAMPs, Sensor proteins, the adaptor protein ASC, pro-caspase-1, IL-1β and IL-18, GSDMD **Non-canonical route**: LPS, K⁺ efflux, caspase-4/5, GSDMD, NLRP3, IL-1β and IL-18 **Caspase-3/8-dependent route**: caspase-8, GSDMD
Necroptosis	ϖ Cell swelling ϖ Organelle breakdown ϖ Plasma membrane rupture ϖ Production of DAMPs	**TNF-α/TNFR1 pathway**: TNF-α binds to TNFR1	**TNF-α/TNFR1 pathway**: TRADD, TRAF2/5, RIPK1, RIPK3, cIAPs, caspase-8, MLKL
PANoptosis	A mixture of morphological features of apoptosis, pyroptosis, and necroptosis ϖ membrane pore formation ϖ Cell shrinkage or swelling ϖ Nuclear fragmentation	Infection with pathogens such as HSV1, IAV, SARS-CoV2, Yersinia, F.Novicida and their PAMPs, heme, cytokines **ZBP1-dependent PANoptosome**: Z-RNA generated by DNA and RNA viruses **AIM2-dependent PANoptosome**: HSV1 or Francisella. novicida (F. novicida) infection **RIPK1-dependent PANoptosome**: Yersinia infection **NLRP3**,** and NLRP12-dependent PANoptosome**: heme and PAMPs	**ZBP1-dependent PANoptosome**: ZBP1, RIPK1 and RIPK3, ASC/NLRP3/CASP1-GSDMD and CASP3/6/7 and MLKL **AIM2-dependent PANoptosome**: AIM2, Pyrin, ZBP1, ASC, caspase-1, caspase-8, RIPK1, RIPK3, and FADD **RIPK1-dependent PANoptosome**: RIPK1 and 3, caspase-8, ASC, NLRP3, and caspase-1 **NLRP3**,** and NLRP12-dependent PANoptosome**: NLRP3, NLRP12, ASC, caspase-8, and RIPK3

*Abbreviation*: *BCL-2* Pro-apoptotic members of the B-cell lymphoma 2, *BAK* BCL2-antagonist/killer 1, *BAX* BCL2-associated X protein, *Apaf-1* Apoptotic protease activating factor-1, *dATP*: deoxyadenosine triphosphate, *FADD* Fas-associated death domain protein, *TRADD* Tumor necrosis factor receptor-associated DEATH domain protein, *PAMP* Pathogen-Associated Molecular Patterns, *DAMP* Damage-Associated Molecular Patterns, *GSDMD* Gasdermin D, *IL-1β *Interleukin-1β, *IL-18* Interleukin-18, *LPS* lipopolysaccharide, *NLRP3* NOD-, LRR-, and pyrin domain-containing protein 3,* GzmA* granzyme A, *GzmB* granzyme B, *PD-L1* Programmed Cell Death Ligand 1, *STAT3* Signal Transducer and Activator of Transcription 3,* TNFα* Tumor necrosis factor α, *TRAF2/5* TNF receptor-associated factor 2/5, *RIPK1* Receptor-interacting protein kinase 1, *cIAPs* cellular inhibitor of apoptosis proteins, *MLKL* Mixed Lineage Kinase Domain-Like, *HSV1* Herpes simplex virus 1, *IAV* Influenza A virus, *PAMP* Pathogen associated molecular pattern, *DAMP* Damage associated molecular pattern, *F. Novicida* Francisella. Novicida,* SARS-CoV-2* Severe acute respiratory syndrome coronavirus 2

### Pyroptosis

A new kind of PCD with lytic and pro-inflammatory characteristics is pyroptosis. While both pyroptotic and apoptotic cells exhibit chromatin condensation and DNA breakage, pyroptotic cells differ from apoptotic cells in that they exhibit pore development, cell swelling, the release of proinflammatory chemicals, and osmotic lysis [39, 40]. Research on pyroptosis advanced rapidly after the identification of GSDMD as a crucial effector protein. This led to the study of many pathways, including granzyme-mediated, canonical, non-canonical, and caspase-3/8-dependent processes [41]. In reaction to stimuli, caspases-1, 3–5, 8 and 11 or granzymes (such as granzyme A/B) may cleave GSDMD, resulting in the formation of large openings in the cell membrane that leak contents, induce pyroptosis, and encourage inflammation [42].

PAMPs and DAMPs initiate intracellular signaling cascades that facilitate inflammasome formation in the canonical pyroptosis pathway [43]. Sensor proteins, pro-caspase-1, and the adaptor protein ASC usually make up these complexes. Upon assembly, caspase-1 becomes activated and subsequently cleaves the proforms of the inflammatory cytokines IL-18 and IL-1β, in addition to GSDMD [44, 45]. Water influx, membrane rupture, cell enlargement, and cell death result from the N-terminal segment of GSDMD inserting into the plasma membrane to create non-selective holes. These GSDMD pores are also responsible for the release of IL-1β and IL-18 [46]. The non-canonical process involves the direct activation of caspase-4/5 by cytosolic lipopolysaccharide (LPS), which cleaves GSDMD and causes pyroptosis [47]. Furthermore, potassium (K⁺) efflux resulting from GSDMD-mediated pore formation facilitates the activation of the NLRP3 inflammasome, leading to the maturation of IL-18 and IL-1β [48]. Inhibition of TAK1 triggers caspase-8, which may cleave GSDMD and start pyroptosis in the caspase-8-dependent pathway [41].

**Fig. 2:**
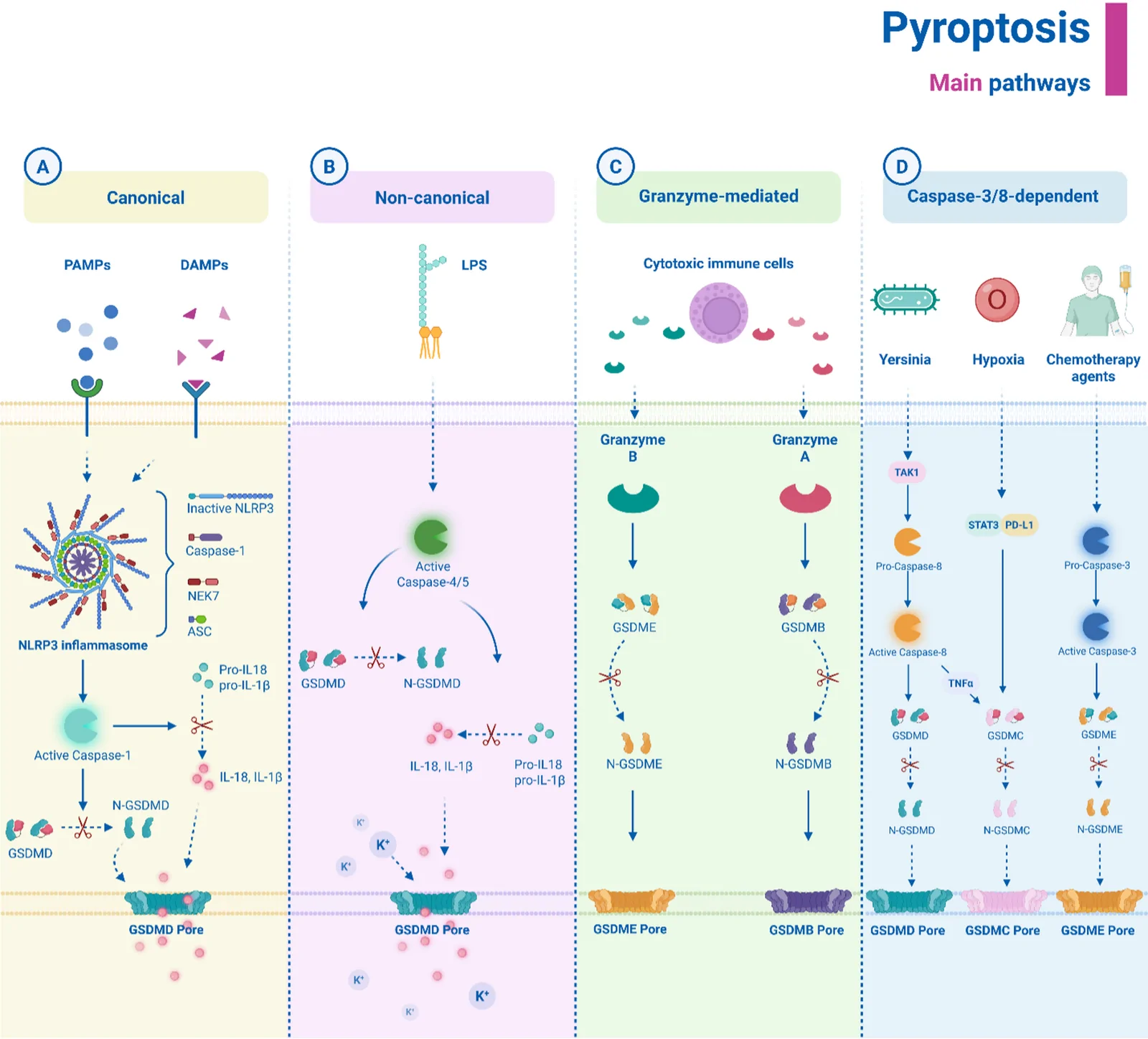
Critical molecular pathways involved in pyroptosis. This figure displays the four principal pyroptotic signaling pathways: (**A**) canonical, (**B**) non-canonical, (**C**) granzyme-dependent, and (**D**) caspase-3/8-dependent. **A** Canonical pathway; PAMP and DAMP perception triggers activation of the NLRP3 inflammasome, which sequesters adaptor protein ASC and pro-caspase-1. Activated caspase-1 cleaves GSDMD, generating N-GSDMD fragments that form membrane pores, allowing for secretion of IL-1β and IL-18 and leading to pyroptotic lysis. **B** The non-canonical pathway is direct intracellular LPS activation of caspase-4/5 (humans) or caspase-11 (mice), proteolytic cleavage of GSDMD and leading to pore formation, which also has the ability to activate NLRP3 inflammasome. **C** In the granzyme-dependent pathway, cytotoxic lymphocytes release granzymes A and B. Granzyme B proteolytically cleaves GSDME, and Granzyme A cleaves GSDMB, to produce pore-forming N-GSDME and N-GSDMB fragments, respectively, to execute pyroptosis. **D** Caspase-3/8-dependent pathway bridges the pyroptotic and apoptotic pathways. Cleavage of GSDME, GSDMD, or GSDMC by stressors such as infection (Yersinia), hypoxia, or chemotherapy agents activates caspase-3 or −8 and triggers inflammatory cell death. In this pathway, transcription factors such as STAT3 and immune checkpoint proteins such as PD-L1 might regulate the expression of GSDMC and thus the pathway choice between apoptosis and pyroptosis. Collectively, these interconnected pathways depict the molecular heterogeneity of pyroptosis and its role in immunity-mediated inflammation and cell death

Pyroptosis is initiated by GzmB-induced activation of caspase-3, leading to the cleavage of GSDME. As an alternative, GzmA cleaves GSDMB, and GzmB may activate GSDME directly without the need for caspase [49]. GSDMC expression is induced by PD-L1 translocating into the nucleus and interacting with phosphorylated STAT3. Caspase-8 cleaves GSDMC in response to TNF-α activation, thereby altering cell death from apoptosis to pyroptosis [41]. The main morphologic features, triggers, and players of pyroptosis are listed in Table 1 and illustrated in Fig. 2.

### Necroptosis

**Fig. 3:**
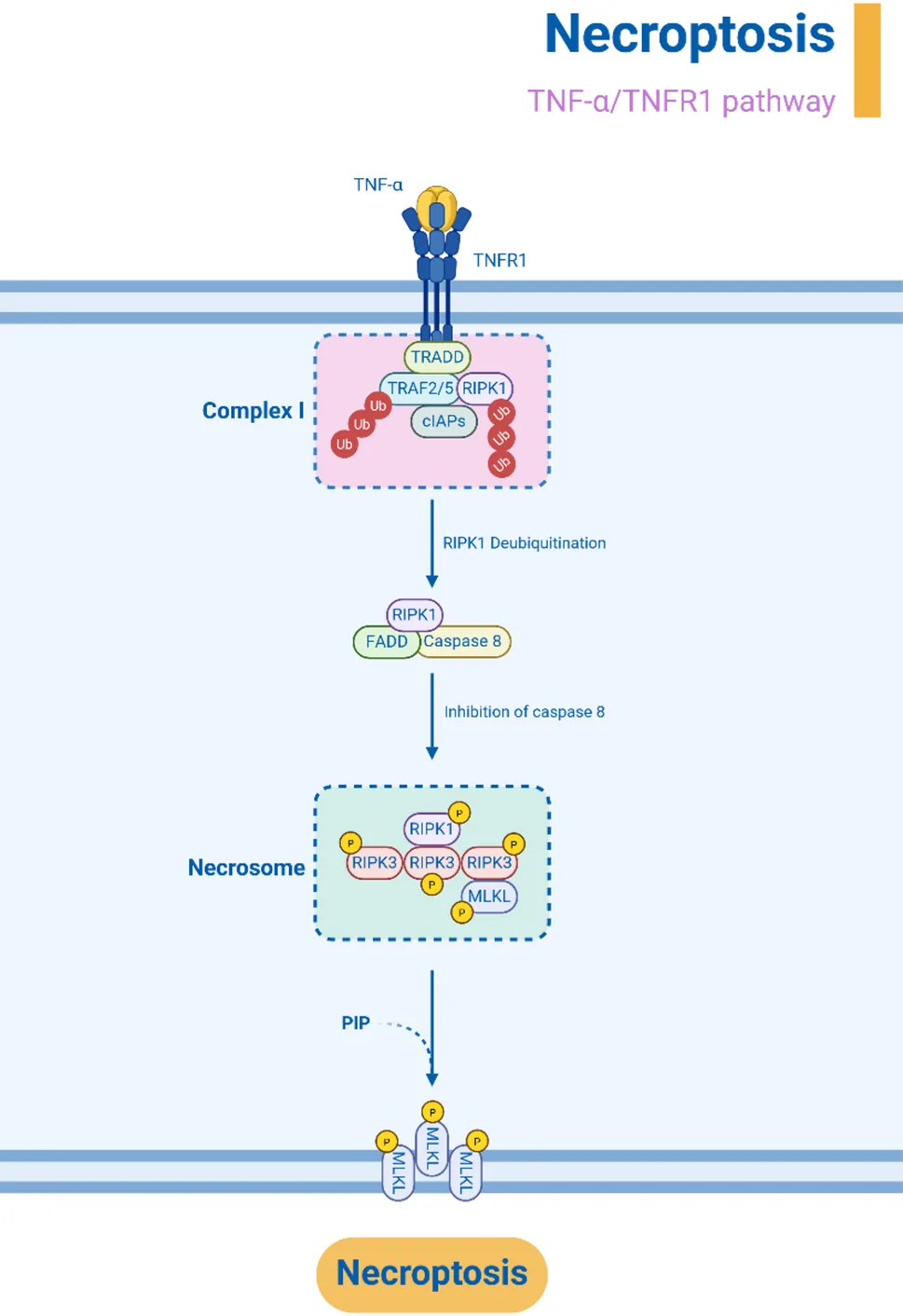
TNF-α/TNFR1-induced necroptosis signaling pathway. The molecular mechanism of necroptosis is illustrated in the diagram, a controlled inflammatory cell death process distinct from apoptosis. Upon binding of TNF-α to its receptor TNFR1, Complex I form on the plasma membrane consisting of TRADD, TRAF2/5, RIPK1, and cIAPs, which ubiquitinate RIPK1 to facilitate cell survival signaling. Deubiquitination of RIPK1 triggers FADD and caspase-8 recruitment to form Complex II. When the function of caspase-8 is inhibited, RIPK1 interacts with RIPK3 through RHIM domains to form the necrosome. Inside the necrosome, RIPK1 and RIPK3 phosphorylate one another, activating phosphorylation of mixed lineage kinase domain-like protein (MLKL). p-MLKL moves towards the plasma membrane and interacts with phosphatidylinositol phosphates (PIPs), disrupting membrane integrity and causing osmotic disruption, swelling, and cell bursting, which are characteristics of necroptosis. This route focuses on the central role of RIPK1, RIPK3, and MLKL in necroptosis mediating inflammatory and reproductive pathologies

Necroptosis is a controlled kind of cell death that resembles necrosis in appearance but functions differently than either necrosis or apoptosis. Cell shrinkage, DNA breakage, and membrane blebbing are the hallmarks of apoptosis, which is caused by caspase activation and lacks an inflammatory component [50]. Necroptosis is characterized by cell swelling, organelle breakdown, and plasma membrane rupture, all of which lead to the production of DAMPs that activate a severe inflammatory response [51]. One of the best-studied pathways that triggers necroptosis is the TNF-α/TNFR1 pathway. Complex I, a signaling complex that includes TRADD, TRAF2/5, RIPK1, and cIAPs, forms when TNF-α binds TNFR1 [52]. On the other hand, RIPK1 dissociates from the membrane complex and associates with RIPK3 upon deubiquitination or when caspase-8 is pharmacologically inhibited [53]. This triggers the formation of a necrosome, after which RIPK3 becomes phosphorylated and phosphorylates MLKL, a crucial executor of necroptosis [53, 54]. The execution phase of necroptosis is triggered when phosphorylated MLKL oligomerizes and is recruited to the plasma membrane, where it binds phosphatidylinositol phosphates and compromises membrane integrity, ultimately leading to membrane rupture, ionic imbalance, cellular swelling, and the release of intracellular contents [55]. Necroptosis is essential for regulating immune responses and preserving tissue homeostasis. Several illnesses, including cancer, inflammatory disorders, and neurological diseases, are associated with the dysregulation of necroptosis [56, 57]. The main morphologic features, triggers, and players of necroptosis are listed in Table 1 and illustrated in Fig. 3.

### PANoptosis

apoptosis, and pyroptosis work together to form the multi-protein PANoptosome complex, the result of PANoptosis [58]. In response to various stressors, including herpes influenza A virus (IAV) and simplex virus 1 (HSV1) infection, the PANoptosome complex assembles several PANoptotic effectors and induces cell death [59]. There are three types of proteins in normal PANoptosome assemblies: (1) sensor proteins (such as NLRP3 and Z-DNA-binding protein 1 (ZBP1/DAI)); (2) adapter proteins (like ASC) that have caspase recruitment domains; and (3) catalytic proteins (like RIPK3, RIPK1, and caspase-1/8) [60]. The ZBP1-dependent PANoptosome and the AIM2-dependent PANoptosome are now considered as the two prototype PANoptosome assemblies [7]. There have also been reports of other types, such as the RIPK1- and NLRP12-PANoptosomes [61, 62]. The creation of PANoptosomes is mediated by or involves a multitude of proteins, and the makeup of PANoptosomes resulting from different circumstances also tends to differ. Through signal transduction, SARS-CoV-2-generated TNF-α and IFN-γ may facilitate the RIPK1/RIPK3/FADD/CASP8 PANoptosome assembly [63], and ZBP1 is most likely implicated in this process [64]. A ZBP1-independent PANoptosome consisting of RIPK1/RIPK3/CASP8 was notably triggered by Yersinia, and its development was associated with the stimulation of TNFR/TLR4 signaling and the inhibition of MAPKs and NF-κB signaling [62]. Additionally, PANoptosis and the inflammasome are closely linked, and the NLRC4 inflammasome plays a significant role in PANoptosis, in addition to the traditional NLRP3 inflammasome [65]. Importantly, most mechanistic evidence supporting PANoptosis originates from infectious diseases, inflammatory disorders, and cancer models. Whether identical PANoptosome complexes assemble in female reproductive tissues under pathological conditions remains to be experimentally confirmed. The main morphologic features, triggers, and players of PANoptosis are listed in Table 1.

## Molecular mechanisms and components of PANoptosome

### ZBP1 PANoptosome

Two Zα domains, which bind nucleic acids, are located at the N-terminus of ZBP1, and two RHIM domains are located in the center of the protein sequence [66]. PANoptosis is caused by the ZBP1 PANoptosome, which causes apoptosis, pyroptosis, and necroptosis by activating the ASC/NLRP3/CASP1-GSDMD, CASP3/6/7, and MLKL pathways [67]. Because of its Zα domains, ZBP1 plays an essential role as an innate immunological sensor for Z-RNA produced by RNA and DNA viruses. The RHIM domains found on ZBP1 allow it to interact with other proteins that possess RHIM, such as RIPK1 and RIPK3. During IAV infection, the ZBP1-RIPK1 complex may regulate TNF-α and IL-6 synthesis and transduce NF-κB signaling [68].

**Fig. 4:**
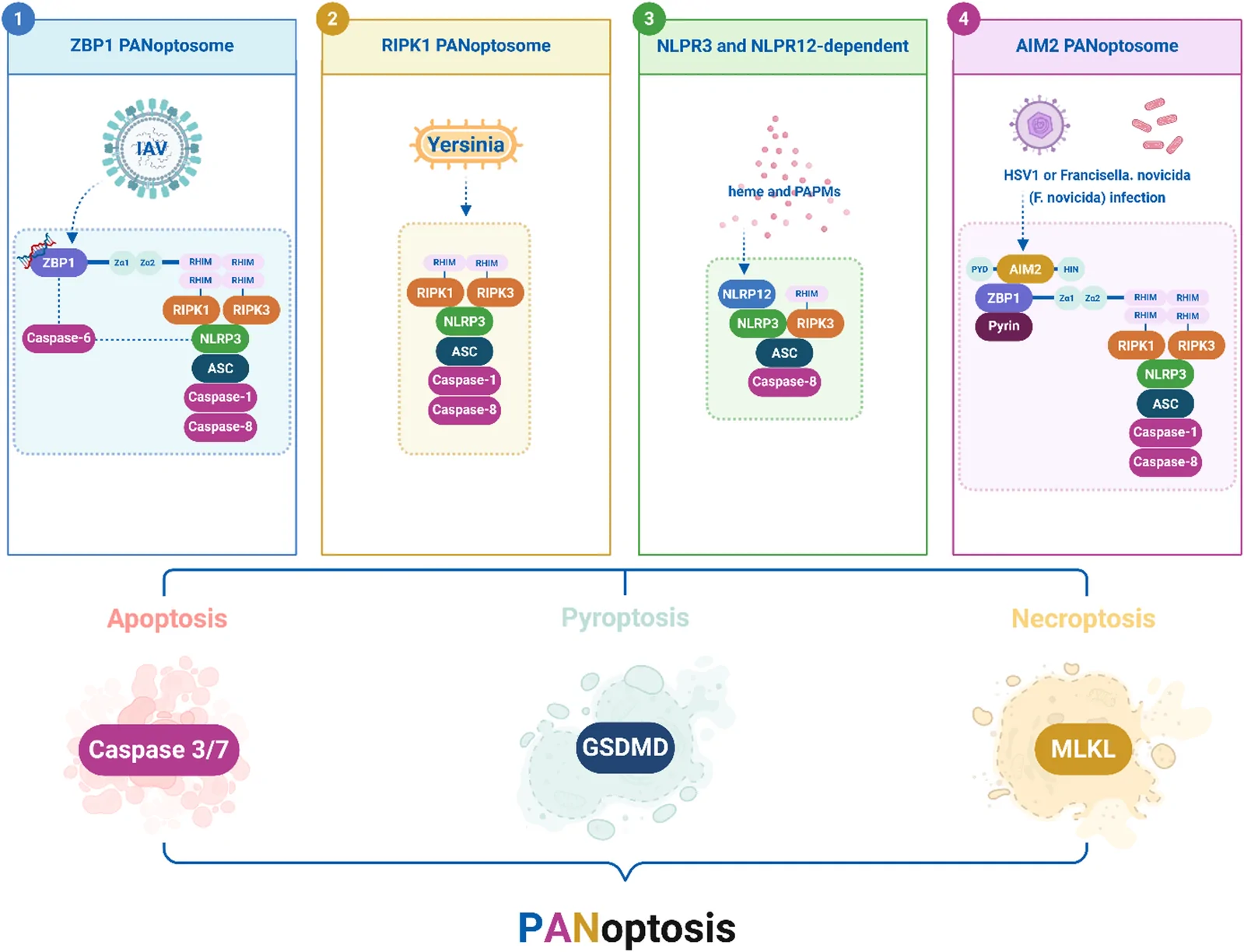
Structure and function of the PANoptosome complex, encompassing apoptosis, pyroptosis, and necroptosis. This figure illustrates the four well-defined PANoptosome complexes that facilitate PANoptosis, a singular cell death mechanism that includes pyroptosis, apoptosis, and necroptosis. 1 ZBP1 PANoptosome: Upon infection with influenza A virus (IAV), ZBP1 engages with RIPK1 and RIPK3 through its RHIM domains, leading to the recruitment of NLRP3, ASC, and caspases-1, −6, and − 8. These complex initiates apoptosis via caspase-3/7, pyroptosis via GSDMD, and necroptosis via MLKL. 2 RIPK1 PANoptosome: Induced during Yersinia infection, this complex comprises RIPK1, RIPK3, ASC, NLRP3, caspase-1, and caspase-8, facilitating PANoptotic cell death in response to microbial pathogens. 3 NLRP3/NLRP12-dependent PANoptosome: Triggered by heme or pathogen-associated molecular patterns (PAMPs), this complex includes NLRP3, NLRP12, ASC, RIPK3, and caspase-8, facilitating inflammatory cell death. 4 The AIM2 PANoptosome; which is activated by HSV-1 or Francisella novicida infection, comprises a complex that includes AIM2, ZBP1, Pyrin, ASC, RIPK1, RIPK3, and caspases-1 and − 8, effectively integrating cell death and inflammatory signaling pathways. The complexes collectively target the apoptosis executioners (caspase-3/7), the pyroptosis executioner (GSDMD), and the necroptosis executioner (MLKL), integrating various programmed cell death pathways into PANoptosis. This collaborative process highlights the intricacies of innate immune signaling and its significance in inflammatory and reproductive diseases

ZBP1, NLRP3, ASC, caspase-8 and 1, RIPK3 and 1 comprise the ZBP1-dependent PANoptosome [69]. Also, caspase-6 binds RIPK3, increasing the number of bridges between ZBP1 and RIPK3 and aiding in complex formation [69]. ZBP1 has the potential to initiate NF-κB and IFN signaling as a very effective DNA sensor in the cytosol [70]. It regulates RIPK1-caspase-8-RIPK3 pathways, which in turn trigger PANoptosis, and acts as a center for regulating inflammatory cell death [71]. The signaling pathway of ZBP1 PANoptosome is illustrated in Fig. 4.

### RIPK1 PANoptosome

RIPK1 controls multiple forms of cell death, TNFR1 signaling, and the production of inflammatory cytokines [72]. A RIPK1-dependent PANoptosome, including RIPK1 and 3, caspase-8, ASC, NLRP3, and caspase-1, is formed in macrophages during Yersinia infection [62]. IRF1 plays a crucial regulatory role in controlling PANoptosis during NLRP12-dependent PANoptosome formation, when NLRP12, caspase-8, ASC, and RIPK3 are all present, and PANoptosis occurs in response to PAMPs and heme [73]. Moreover, it was found that IAV infection did not cause the death of fibroblasts or bone marrow-derived macrophages (BMDMs) lacking RIPK3 [68]. This clearly shows how crucial RIPK3 is to the ZBP1 PANoptosome, which is primarily responsible for stimulating the NLRP3 inflammasome, CASP8/3/7 activation, and MLKL phosphorylation. Additionally, simultaneous administration of MLKL and CASP8 inhibitors prevented IAV infection-induced apoptosis, pyroptosis, and necroptosis in WT BMDMs [74]. The signaling pathway of RIPK1 PANoptosome is illustrated in Fig. 4.

### NLRP3, and NLRP12-dependent PANoptosome

With IAV infection, pyroptosis (one arm of PANoptosis) is mainly caused by the NLRP3 inflammasome. Research shows that mice deficient in CASP1 died more quickly after infection with IAV, and that host defense against IAV relies on activation of NLRP3 inflammasomes [75, 76]. It’s worth noting that NLRP3 suppression at the end of infection reduced the severity of IAV infection [77]. Because of this, IAV resistance was at least largely caused by early-stage NLRP3 inflammasome activation, and ZBP1 deletion completely eliminated this effect [78]. In the case of vesicular stomatitis virus and other pathogen infections, ZBP1 might not be required for the activation of the NLRP3 inflammasome [68]. This implies that the PANoptosome’s ZBP1-dependent assembly is specific to infectious environments, such as those caused by IAV, where it coordinates the activation of necroptosis, apoptosis, and pyroptosis. Furthermore, it has been suggested that NLRP3 may also function as a scaffold protein within the PANoptosome, helping to form a multiprotein death-inducing complex comprising MLKL, RIPK3, and caspase-8 [65].

NLRP12, a NOD-like receptor (NLR) family member, is tightly associated with innate immunity and inflammatory disorders [79]. According to earlier research, NLRP12 acts as a cytosolic PRR during P. chabaudi or Yersinia pestis infections, acting as an inflammasome sensor to trigger pyroptosis [79, 80]. In addition to triggering the inflammasome, new research indicates that NLRP12 may also function as a PANoptosome sensor, causing PANoptosis when heme and PAMPs are co-stimulated [61]. Through IRF1, upstream TLR2 and TLR4 regulate NLRP12 expression in this process [81]. The signaling pathway of NLRP3 and NLRP12-dependent PANoptosome is illustrated in Fig. 4.

### AIM2 PANoptosome

The AIM2-dependent PANoptosome consists of AIM2, ZBP1, Pyrin, caspase-1, RIPK3, caspase-8, RIPK1, ASC, and FADD, involved in inflammatory cell death [82]. Evidence suggests that AIM2 regulates pyrin and ZBP1 to enhance PANoptosis and inflammatory responses in HSV-1 and F. novicida infections [82]. AIM2 has a PYD at the N-terminus and HIN domain at the C-terminus, allowing for DNA sensing and recruiting ASC and caspase-1 once activated [83]. FADD serves as an adaptor protein for caspase-8 in the RIPK1-caspase-8-FADD axis, which also facilitates inflammatory signaling [84]. Caspase-8 and FADD function in this manner to inhibit spontaneous necroptosis by repressing ZBP1 and necroptotic signalling pathways [85], while FADD limits ZBP1- and TNFR1-mediated downstream inflammation by limiting caspase-8–GSDMD pyroptosis and MLKL necroptosis in epithelial cells [86]. The lack of ZBP1 diminished the impact of AIM2. Following infection with HSV1 or F. novicida, Zbp1^–/–^ BMDMs showed reduced activation of CASP8/3/7, CASP1/GSDMD/GSDME, and RIPK3/MLKL in comparison to WT BMDMs, while Aim2^–/–^ and Zbp1^–/–^ BMDMs showed total inactivation [82]. Remarkably, HSV-1 and F. novicida enable the formation of PANoptosomes with a range of sensors by inhibiting Rho-GTP activity, attracting the ZBP1 Zα domain, and activating AIM2 [82]. Furthermore, caspase-8 can support inflammasome action independently of FADD [84]. IRF1 also regulates AIM2-dependent PANoptotic responses during Francisella novicida and HSV-1 infection; IRF1-deficient bone marrow-derived macrophages had reduced death and PANoptotic markers [87]. The signaling pathway of NLRP3 and AIM2 PANoptosome is illustrated in Fig. 4.

## Regulation of PANoptosis

In the same way that apoptosis, pyroptosis, and necroptosis all include genetic determinism, PANoptosis involves regulated cell death in order to preserve the organ or tissue structure. Therefore, controlled cell death is essential for immune protection. In general, there are three primary steps involved in regulating PANoptosis: (1) identifying PAMPs or DAMPs; (2) controlling downstream effector functions, and (3) altering the PANoptosome components both before and after translation [65]. On the other hand, tissue damage may result from poorly controlled cell death. For example, co-activation of TNF-α and IFN-γ during SARS-CoV-2 infection may result in PANoptosis, which causes deadly cytokine shock in mice; survival was increased by suppressing PANoptosis [63]. Therefore, it’s critical to control PANoptosis properly to preserve immunological balance and reduce host damage.

One crucial transcriptional regulator of PANoptosis is interferon regulatory factor 1 (IRF1), which coordinates the production of many molecules involved in cell death [88]. ZBP1 is upstream of IRF1, which, during viral infections like IAV, promotes ZBP1 transcription [89]. In cultures devoid of IFNAR1/2 or downstream IFN-signaling proteins such as IRF9 or STAT1, ZBP1 is not expressed in response to IFN signaling [78]. IRF1 deletion inhibits pyroptosis, apoptosis, and necroptosis by lowering ZBP1 expression and further activating PANoptosis mediators [89, 90]. IRF1 knockout reduced ZBP1 only partially during IAV infection, suggesting that redundancies or compensation are occurring through other sensing pathways that have not yet been identified, despite RIG-I being implicated as a potential upstream sensor mediating this response in BMDMs [78].

Assembly of PANoptosomes is regulated by other factors unrelated to protein synthesis. Deaminase of Adenosine Restricting ZBP1-mediated PANoptosis and promoting tumor growth are achieved by acting on RNA 1 (ADAR1), particularly the p150 isoform [91]. In the cytoplasm, ADAR1-p150 competes with RIPK3 for binding with ZBP1 and has a Zα domain that is acetylated by interferon. Viral entities also affect PANoptosis, even though host cellular regulators do. One known trigger of NLRP3 inflammasome activation is H⁺ efflux from the Golgi apparatus, which can be promoted by the ion channel activity of the influenza virus’s M2 matrix protein [92]. Furthermore, viral proteins such as NS1 and PB1-F2 function as modulators of the NLRP3 inflammasome, providing further evidence of the connection between pathogen sensing and PANoptotic cell death [93].

## Regulated cell death pathways in female reproductive disorders

### Endometriosis

When endometrial cells are seen outside of the uterus, it is known as endometriosis, an estrogen-dependent condition [94]. Endometriosis affects 190 million women globally and affects about 10–15% of reproductive-age women [95]. The process of cellular death in PCD or RCD is highly organized and involves specific molecular pathways and signaling cascades. Apoptosis, necroptosis, pyroptosis, and autophagy are all parts of this evolutionary process that has been passed down over many generations [96, 97]. When everything is working as it should, PCD helps keep tissues in a stable state by removing damaged, aberrant, or old cells. But pathological cell death can occur when PCD is abnormally activated or inhibited in specific disease situations.

Clinical studies of ectopic endometrial tissues obtained from patients with endometriosis have demonstrated abnormal patterns of regulated cell death. The onset and progression of this disease may have close ties to this occurrence [98]. Ectopic myomas are more likely to form because endometrial cells in these patients have a lower apoptosis rate, which means that exfoliated cells are able to elude the body’s clearance processes and continue to proliferate in ectopic lesions [99]. Furthermore, endometrial cells from EM patients undergo autophagy at a much lower rate than normal endometrial cells in situ [100]. Endometrial cells may be able to persist in ectopic lesions after immune system identification decreases autophagy and death. Research has shown that cells derived from the endometrium, whether they are located in the ectopic or eutopic endometrial space, have a better chance of survival in women who have endometrial cancer [99, 101]. Studies using human eutopic and ectopic endometrial stromal cells have demonstrated that endometrial cells from EM patients are less apoptotic than healthy control cells. Previous studies have demonstrated that compared to normal endometrial cells and EuESCs, EESCs express higher levels of the anti-apoptotic genes Bcl-2 and Bcl-Xl. Ectopic endometrial cells have a better chance of surviving when their expression is up because these genes prevent cell death. Another piece of evidence supporting EESCs’ resistance to apoptosis is the substantial downregulation of caspase-3, an apoptosis-related gene [102].

Inflammatory and immunological responses, tissue homeostasis maintenance, and necroptosis signaling are just a few of the physiological processes that are intricately connected [103]. Evidence suggests that EM pathogenicity, as measured by inflammation and immune cell recruitment, is correlated with the expression of genes relevant to necroptosis [100, 104]. Interactions among endometrial, epithelial, and stromal cells as well as other inflammatory mediators describe endometriosis, an inflammatory illness. Evidence suggests the NLRP3 inflammasome may have a role in EM development and progression [105]. The pyroptotic pathway mediated by NLRP3/caspase-1/IL-1β is linked to endometriosis, as demonstrated by Hang et al. [106]. Clinical studies have reported increased NLRP3 expression in endometriotic tissues and serum samples from patients with endometriosis [107]. Many inflammatory molecules, including IL-1β, are produced by the NLRP3 inflammasome and are associated with the pathogenicity and progression of EM [108]. It is believed that the aberrant activation of pyroptosis in the endometrial cell (EC) endometrium is the source of the elevated inflammation observed in endometriosis, as the NLRP3-mediated pyroptosis pathway generates significant amounts of interleukin-1β. There is evidence that the endometrium of ECs exhibited more fibrosis and pyroptosis, and that reducing pyroptosis, IL-1β production, and TGF-β1-mediated fibrosis was achieved by suppressing NLRP3 in embryonic stem cells [109].

**Fig. 5:**
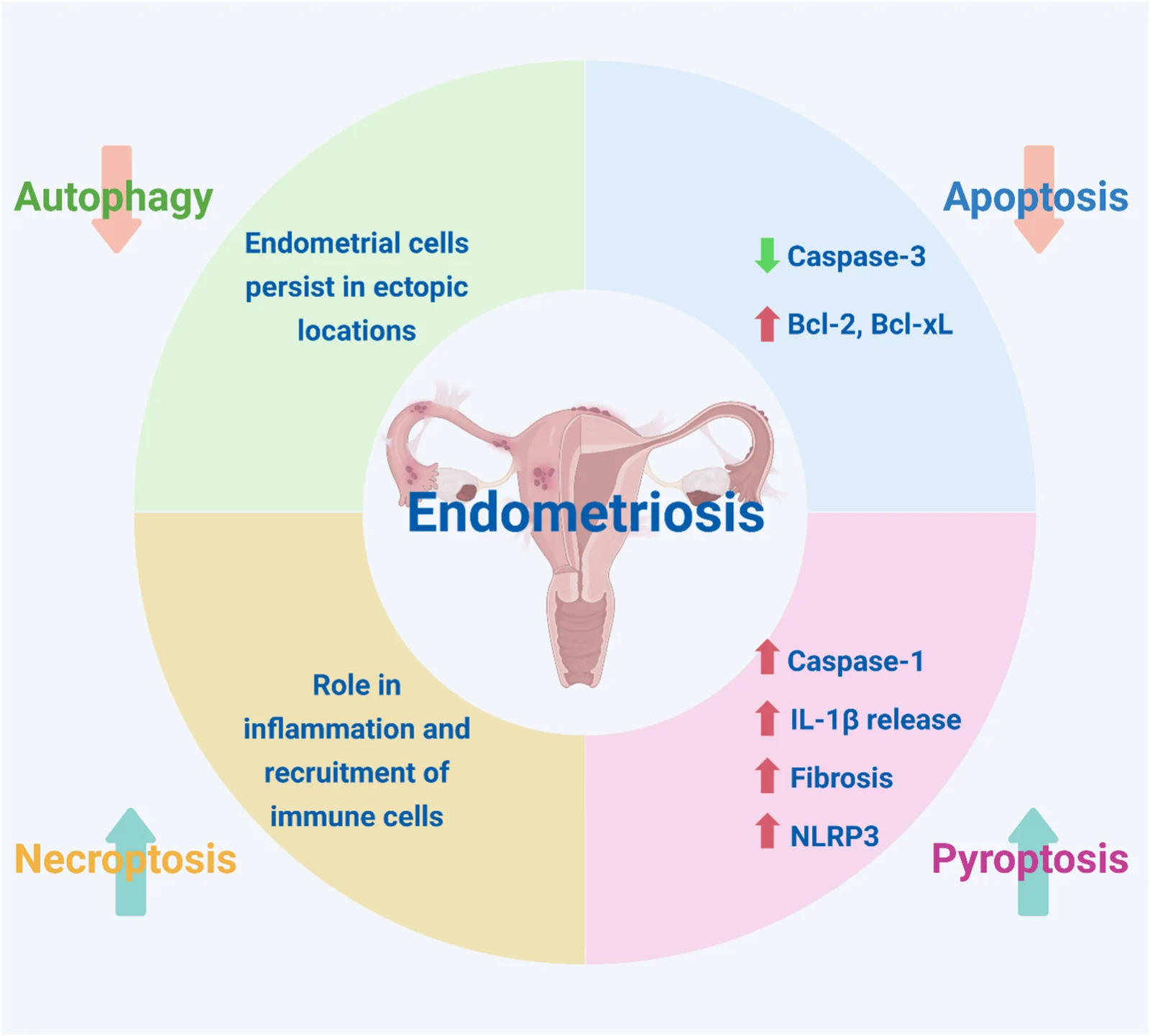
The role of programmed cell death pathways in endometriosis. This figure illustrates dysregulation of several programmed cell death (PCD) pathways (autophagy, apoptosis, necroptosis, and pyroptosis) in endometriosis pathogenesis. Autophagy (↓): Reduced activity of autophagy favors survival of endometrial cells at ectopic sites. Apoptosis (↓): A significant decrease in apoptosis in ectopic endometrial stromal cells (EESCs) is observed by the decrease in caspase-3 expression and increase in anti-apoptotic proteins Bcl-2 and Bcl-xL, promoting cell survival and proliferation in ectopic locations. Necroptosis (↑): Involvement in inflammation and immune cell recruitment identifies this pathway as a candidate in the chronic inflammatory environment present in endometriosis. Pyroptosis (↑): Upregulation of NLRP3 inflammasome, caspase-1, and release of IL-1β have been associated with increased fibrosis and inflammatory responses in the ectopic endometrium. Together, dysregulation of these modes of cell death disrupts normal patterns of endometrial cell turnover and promotes survival of ectopic cells, inflammation, and fibrosis, and finally endometriosis progression

In an LPS-induced murine model of endometritis and complementary in vitro experiments using primary mouse endometrial epithelial cells, total flavonoids of C. chinense (TFC) reduced inflammasome activation and pyroptosis through inhibition of the TLR4/NF-κB/NLRP3 axis [110]. Additionally, using a murine model of LPS-induced endometritis, Gao et al. showed that melatonin reduced LPS-induced endometritis by inhibiting NLRP3 inflammasome activation through autophagy [111]. These results demonstrate that the NLRP3 inflammasome is critical for endometritis development and progression and that endometrial inflammation treatment options may include focusing on this inflammasome. Involvement of different forms of PCD in the pathogenesis of endometriosis are listed in Table 2 and illustrated in Fig. 5.

**Table 2:** Representative studies investigating regulated cell death pathways in female reproductive disorders

Disorder	Experimental model	Cell death pathway	Key molecular mediators	Principal findings	Level of evidence	Study (Ref.)
Endometriosis	Human endometriotic tissues	Pyroptosis	NLRP3, Caspase-1, IL-1β	Demonstrated activation of the NLRP3/caspase-1/IL-1β axis in endometriosis.	Human	[106]
Endometriosis	LPS-induced mouse endometritis + primary endometrial epithelial cells	Pyroptosis	TLR4, NF-κB, NLRP3	TFC inhibited inflammasome activation and pyroptosis.	Animal + in vitro	[110]
Endometriosis	Mouse model	Pyroptosis	NLRP3, Autophagy	Melatonin attenuated endometritis through inhibition of NLRP3 activation.	Animal	[111]
PCOS	Human transcriptomic dataset	Necroptosis	IL-33, BCL2, TNFSF10	Established a necroptosis-related risk signature for PCOS.	Human (bioinformatics)	[112]
PCOS	Testosterone-induced mouse model + granulosa cells	Pyroptosis	NLRP3, Caspase-1, GSDMD	Hyperandrogenism activated NLRP3-mediated pyroptosis.	Animal + Human + in vitro	[113]
PE	HTR8/SVneo cells + placental tissues	Apoptosis	IFN-γ, JAK1, STAT1, SOCS1	IFN-γ impaired trophoblast invasion through JAK1/STAT1 activation.	Human + in vitro	[114]
PE	Human placentas + hypoxic trophoblasts	Necroptosis	RIPK1, RIPK3, MLKL, SIRT3	Necroptosis contributed to trophoblast dysfunction in PE.	Human + in vitro	[115]
RPL	Human serum and placenta	Pyroptosis	NLRP3, Caspase-1, IL-18	Increased inflammasome activation in women with RPL.	Human	[116]
RPL	Murine spontaneous abortion model	Pyroptosis	AIM2, ASC, Caspase-1, NLRP3	Inflammasome activation associated with fetal loss.	Animal	[117]
POI	Cyclophosphamide-induced mouse model	Pyroptosis	NLRP3, GSDMD	Leonurine hydrochloride alleviated ovarian injury by suppressing pyroptosis.	Animal	[118]
POI	Mouse model	Proposed PANoptosis	NLRP3, RIPK3, CASP8	Suppressed molecular signatures consistent with PANoptosis; canonical PANoptosome not confirmed.	Animal (emerging evidence)	[119]

*Abbreviations*: *PCOS* Polycystic ovary syndrome, *PE* Preeclampsia, *RPL* Recurrent pregnancy loss, Premature ovarian insufficiency (POI); *JAK1* Janus Kinase 1, *BCL-2* Pro-apoptotic members of the B-cell lymphoma 2, *GSDMD* Gasdermin D, *IL-1β* interleukin-1β, *IL-18* interleukin-18, *NLRP3* NOD-, LRR-, and pyrin domain-containing protein 3, *STAT1* Signal Transducer and Activator of Transcription 1, *RIPK1* receptor-interacting protein kinase 1, *MLKL* Mixed Lineage Kinase Domain-Like, *NF-κB* Nuclear factor kappa-light-chain-enhancer of activated B cells, *IFNγ* Interferon γ, *MAPK* Mitogen-Activated Protein Kinase

### Polycystic ovary syndrome

About 5–20% of reproductive-aged women suffer from polycystic ovarian syndrome (PCOS), an endocrine reproductive condition. Features in the areas of reproduction, metabolism, and hormones include oligo-ovulation, infertility, obesity, hyperinsulinemia, type 2 diabetes, and cardiovascular disease [120].

One of the major contributors to polycystic ovary syndrome (PCOS) could be the apoptosis of ovarian granulosa cells (GCs), which causes oocyte apoptosis and follicular atresia [121]. Some have proposed a relationship between GC apoptosis and negative reproductive consequences, such as problems with fertilization, oocyte maturation, embryo development, and pregnancy outcomes [122]. In an in vitro study using human granulosa HO23 cells, DHEA reduced starvation-induced apoptosis and necroptosis by preserving mitochondrial function, decreasing mitochondrial ROS generation, and maintaining mitochondrial membrane potential [123]. In a study by Mingming W. and colleagues, a necroptosis-based risk model was constructed to assess PCOS progression. The four hallmark genes associated with necroptosis were IL-33, PYMG, BCL2, and TNFSF10, which were discovered by algorithmic and machine learning research. Other substances that were being studied as potential treatments for polycystic ovary syndrome were resveratrol, quercetin, tretinoin, and curcumin [112].

Hypotheses have been advanced that NLRP3 inflammasome production, inflammatory mediator release, and low-grade inflammation in PCOS animals are all driven by hyperandrogenism [124]. In PCOS, anovulation and follicular dysfunction are tightly linked to ovarian fibrosis. Ovarian fibrosis is worsened in PCOS mice when the NLRP3 inflammasome is activated. Therefore, a possible therapeutic target for ovarian fibrosis prevention could be the NLRP3 inflammasome [113]. When the NLRP3 inflammasome is activated, caspase-1 is activated, leading to an increase in the synthesis of mature IL-18 and IL-1β. There are a number of reproductive processes that these cytokines regulate, including ovarian steroidogenesis and the maturation of ovarian follicles [125].

**Fig. 6:**
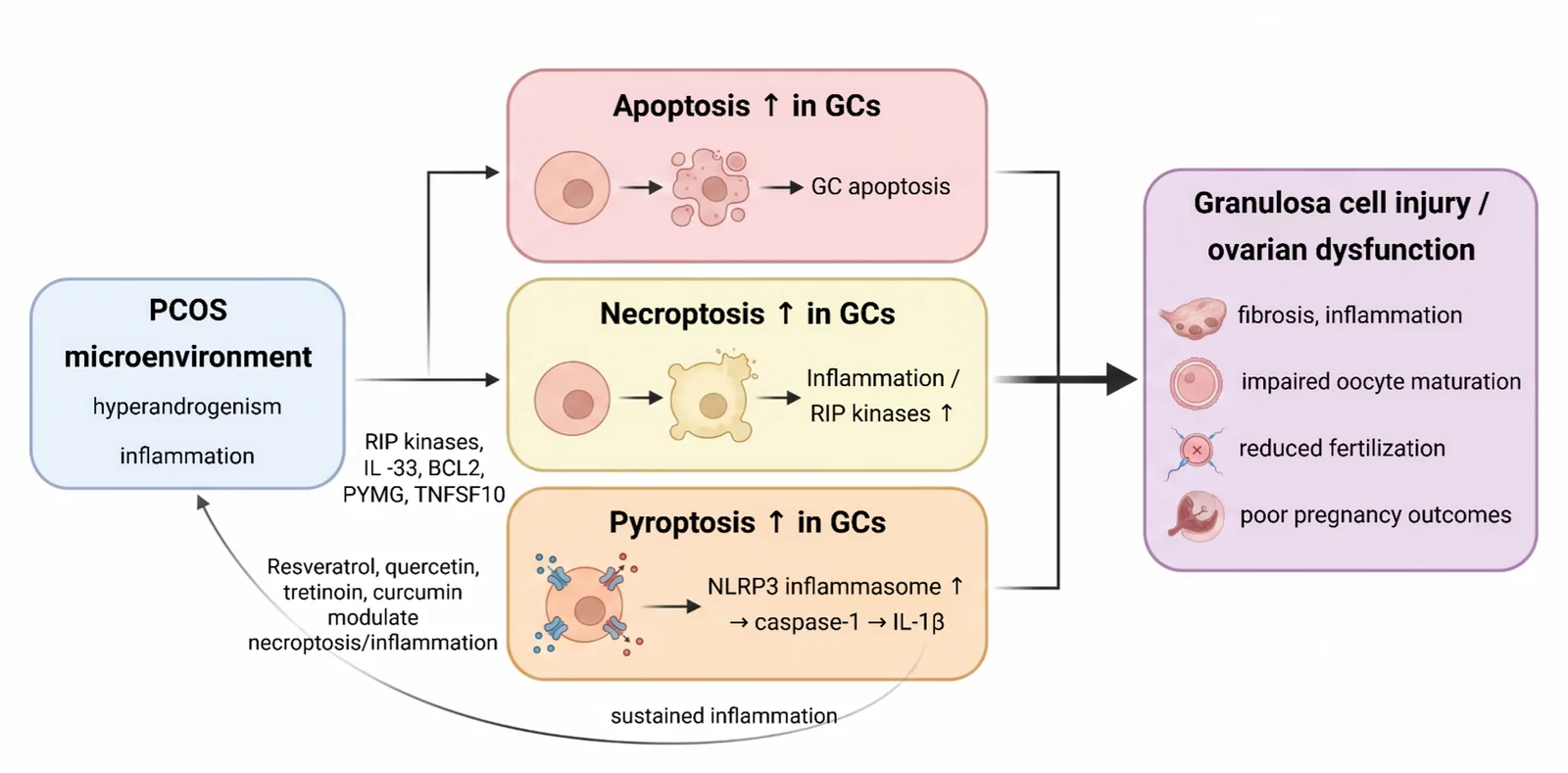
Upregulation of cell death pathways in polycystic ovary syndrome (PCOS). The diagram unifies the heightened programmed cell death pathways (apoptosis, necroptosis, and pyroptosis) in PCOS pathogenesis and their effects on ovarian function. Apoptosis (↑): Enhanced apoptosis of granulosa cells (GCs) leads to impaired oocyte maturation, reduced fertilization, and poor pregnancy outcomes. Necroptosis (↑): Inflammation stimulates necroptosis in GCs by mechanisms involving RIP kinases and necroptosis-related genes such as IL-33, BCL2, PYMG, and TNFSF10. This mechanism results in granulosa cell injury and ovarian dysregulation. The therapeutic agents such as resveratrol, quercetin, tretinoin, and curcumin have been implicated to modulate necroptosis and inflammation in PCOS. Pyroptosis (↑): Hyperandrogenism induces NLRP3 inflammasome activation, which leads to increased caspase-1 and IL-1β release, increased inflammation, and fibrosis of the ovaries, thereby decreasing ovarian function. Overactivation of pyroptosis generates a feedback loop that sustains inflammation and ovarian damage. Together, these dysregulated cell death mechanisms are causally responsible for the inflammatory and fibrotic processes that result in the reproductive and metabolic aberrations of PCOS

Serum steroid hormone levels are associated with various inflammatory indicators, suggesting that immunological activation is a characteristic of PCOS [126]. In a testosterone-induced mouse model of PCOS, hyperandrogenism activated NLRP3 inflammasome signaling and promoted granulosa cell pyroptosis [113]. Consistent with these findings, in vitro experiments demonstrated that NLRP3 inhibition attenuated pyroptosis, while clinical studies of GCs from women with PCOS showed increased expression of caspase-1, GSDMD, and NLRP3. Additionally, GCs from PCOS patients exhibited a substantial upregulation of pyroptosis-related genes and proteins, such as caspase-1, gasdermin D, and NLRP3 [113, 127]. This vicious cycle begins with overactivated pyroptosis, which in turn releases inflammatory molecules that increase inflammation, disturb ovarian function, and worsen the development of PCOS [128]. Involvement of different forms of PCD in the pathogenesis of PCOS are listed in Table 2 and illustrated in Fig. 6.

### Preeclampsia (PE)

A major cause of maternal and perinatal morbidity, PE affects around 5–7% of all pregnancies. PE is linked to high blood pressure after the 20th week of pregnancy, impaired kidney function, and newborns that are undersized for their gestational age. Other complications include renal failure, eclampsia, edema, liver failure, and HELLP syndrome (hemolysis, increased liver enzymes, and low platelets) can worsen the PE-associated pathophysiology [129]. Uneven trophoblast invasion and aberrant spiral artery remodeling are strongly linked to PE; however, the exact cause is still not fully known. During the early stages of gestation, villous cytotrophoblasts have an increase in apoptosis and inflammatory responses, which in turn increases the risk of PE [130]. In an in vitro study using HTR-8/SVneo trophoblast cells, IFN-γ activated the JAK1/STAT1 signaling pathway, suppressed trophoblast migration and invasion, and promoted apoptosis. Consistent with these findings, analysis of placental samples from women with PE demonstrated increased expression of IFN-γ and SOCS1, supporting the clinical relevance of this pathway [114]. Furthermore, oxidative stress is linked to the development of PE. Evidence suggests that NF-kB and p38 MAPK pathways are activated by an excess of placental ROS. Patients with preeclampsia have dysregulated apoptosis due to increased intracellular ROS levels that inhibit SIRT1 action in the placenta [131].

The impact of pyroptosis on PE is extensive. The expression of the NLRP3 inflammasome is higher in the placenta and peripheral blood of preeclamptic pregnancies compared to healthy pregnancies [132]. In response to ongoing inflammation in endothelial cells, various inflammatory mediators are released, which triggers the activation of NLRP3. This in turn releases IL-1β and IL-18 through the pores created by GSDMD, leading to cell death and subsequently PE. HDAC2 down-regulation in pre-eclamptic individuals is associated with elevated levels of FOXO3 and PERK, IL-18 and IL-1β, which in turn hinder cell migration, invasion, and proliferation, and eventually cause trophectodermal pyroptosis [133]. In addition to these, pyroptosis is induced by poorly functioning trophoblast cells, mitochondrial structure, and increased but inefficient glycolysis in PE patients [134]. This may be linked to TLR4/NF-kB activation.

**Fig. 7:**
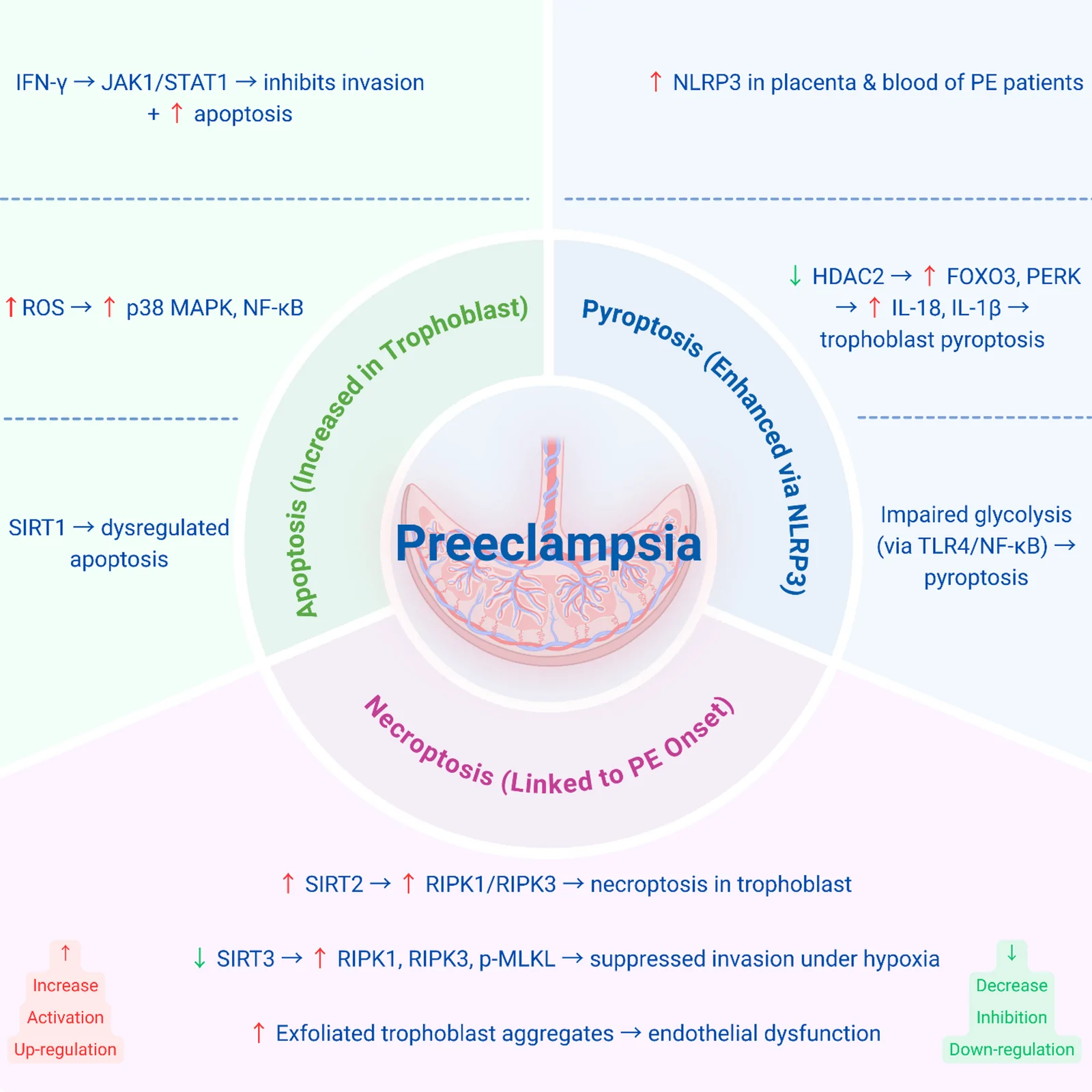
Mechanistic role of apoptosis, pyroptosis, and necroptosis in preeclampsia (PE) pathophysiology. This figure illustrates the interlacing pathways of cell death culminating in preeclampsia, with downstream effects and molecular mediators of trophoblast dysfunction shown. Apoptosis (green segment): Elevated interferon-γ (IFN-γ) activates the JAK1/STAT1 pathway, with reduced trophoblast invasion and enhanced apoptosis. Elevated reactive oxygen species (ROS) activate the p38 MAPK and NF-κB pathways, whereas SIRT1 downregulation leads to inappropriate apoptosis and oxidative placental damage. Pyroptosis (blue region): Maternal and placental circulation NLRP3 inflammasome activation enhances pyroptotic cell death. Reduced expression of HDAC2 activates FOXO3 and PERK signaling to induce IL-18 and IL-1β secretion and trophoblast pyroptosis. Additionally, TLR4/NF-κB-dependent impairment of glycolysis exacerbates pyroptotic injury in trophoblasts. Necroptosis (pink region): The elevated expression of SIRT2 initiates RIPK1/RIPK3-dependent necroptosis, while SIRT3 downregulation upregulates RIPK1, RIPK3, and p-MLKL, suppressing trophoblast invasion under hypoxia. Exfoliated trophoblast aggregates due to necroptotic damage are responsible for endothelial dysfunction, a characteristic of PE. In aggregate, this figure illustrates that disturbed activation of apoptotic, pyroptotic, and necroptotic pathways in placental tissues drives inflammatory injury and trophoblast dysfunction, leading to PE initiation and development

Necroptosis is known to occur during early placentation, and new evidence suggests a strong connection between the two conditions and the start of PE [135]. Deacetylation and activation of RIPK1 occur during the creation of the RIPK1-RIPK3 complex, a process that is NAD-dependent and SIRT2-dependent [136]. A number of different compartments within the placenta, such as decidual cells, cytotrophoblasts, vascular endothelial cells, and amniotic epithelium, have been found to contain SIRT2 [137]. An increase in SIRT2 levels in the placenta has the ability to mediate necroptosis and the loss of trophoblast cells by initiating the development of the RIPK1-RIPK3 complex and the subsequent transmission of death signals. There is evidence that patients with PE have elevated levels of these exfoliated aggregates of trophoblast cells in their peripheral blood, which might cause malfunction of the placental endothelium cells and possibly induce PE [138]. In a study conducted by Yu H and colleagues, it was discovered that PE patients with down-regulated placental SIRT3 had an increase in the expression of RIPK1, RIPK3, and p-MLKL. These genes are involved in promoting necroptosis and suppressing trophoblast invasion and migration when oxygen levels are low [115]. One possible mechanism by which necroptosis promotes PE is by inducing an inflammatory response and immune infiltration; Lidan He et al. found a moderate relationship between the two [139]. It is concluded that necroptosis in the placenta is regulated by multiple functional proteins and immune factors, thereby causing placental dysfunction in distinct ways. Despite growing evidence linking inflammasome-mediated cell death to PE, most mechanistic insights have been derived from trophoblast cell lines and animal models. Furthermore, it remains uncertain whether dysregulated cell death represents a primary pathogenic event or a secondary consequence of placental dysfunction. Involvement of different forms of PCD in the pathogenesis of PE are listed in Table 2 and illustrated in Fig. 7.

### Recurrent pregnancy loss (RPL)

One of the most common complications of pregnancy is RPL, which is defined as two or more failed pregnancies in a row before 24 weeks of gestation. Roughly 3% of pregnancies are affected [140]. Apoptosis, excessive angiogenesis, and invasion are proposed as major pathogenic components of immune-related miscarriages. Excess decidual cell apoptosis and maternal immunological rejection of the embryo can result from impaired fetal-maternal immune responses [141]. During physiological gestation, there is an immunological equilibrium at the fetal-maternal interface. When this balance is disrupted, a shift toward inflammation occurs, and the maternal and fetal compartments become mutually exclusive, leading to fetal loss [142].

As a central element of the inflammatory network, the NLRP3 inflammasome participates in the development of various diseases by regulating multiple inflammatory pathways, but few studies have examined NLRP3 and RPL. Evidence suggests that NLRP3 expression is dramatically upregulated during the endometrial secretory phase in RPL patients [143]. Additionally, research has shown that the decidual and villous tissues of RPL patients had elevated levels of NLRP3 and IL-1β [144]. It has also been shown that, in patients with RPL, abnormal inflammatory responses might be mediated by the NLRP3 inflammasome at the fetal-maternal interface, resulting in reduced trophoblast activity, enhanced macrophage function, and subsequent embryo implantation failure [144]. In a study by Omeljanuik et al., higher serum levels of NLRP3 and IL-18 were observed in RPL patients compared with healthy controls. They also found and increased placental expression of IL-1β, IL-18, and Caspase-1 protein in women who had experienced miscarriage as compared to healthy controls [116].

Both necrotic cells and immune cells can actively secrete HMGB1, which is then discharged to the extracellular matrix. Sterile inflammatory reactions can be set off by this protein. There have been reports of elevated HMGB1 levels in both the maternal-fetal interface and peripheral blood of RPL patients [145]. Additionally, it has been shown that at the maternal-fetal interface, HMGB1-RAGE/TLR2/TLR4-NFkB signaling pathways are engaged in individuals with idiopathic RPL. Furthermore, administration of low-dose aspirin has been shown to reduce HMGB1 expression in subjects with RPL [146]. Infected placental trophoblasts undergo pyroptosis due to apoptotic pathways activated by caspase-8 [147]. Research has shown that placental cells undergo pyroptosis when infected with the Zika virus. This process is initiated when RIG-I recognizes the viral genome, which in turn causes the release of TNF-α. The placental pyroptosis functional factor, GSDME, is activated when this cytokine triggers caspase-87 and caspase-3 cleavage [148]. Beyond inflammasome-mediated inflammation, accumulating evidence indicates that immune dysregulation, characterized by an imbalance of pro-inflammatory cytokines, altered Th1/Th2 responses, and impaired maternal-fetal immune tolerance, contributes substantially to the pathogenesis of RPL [149]. A clinical study in women with RPL further demonstrated dysregulation of immune- and hypoxia-related pathways, including reduced CD39 expression together with increased CD73 and HIF-1α levels in PBMCs. These alterations were accompanied by an increased Th17/Treg ratio, suggesting that immune imbalance and hypoxia-associated signaling contribute to the inflammatory microenvironment underlying RPL [14].

**Fig. 8:**
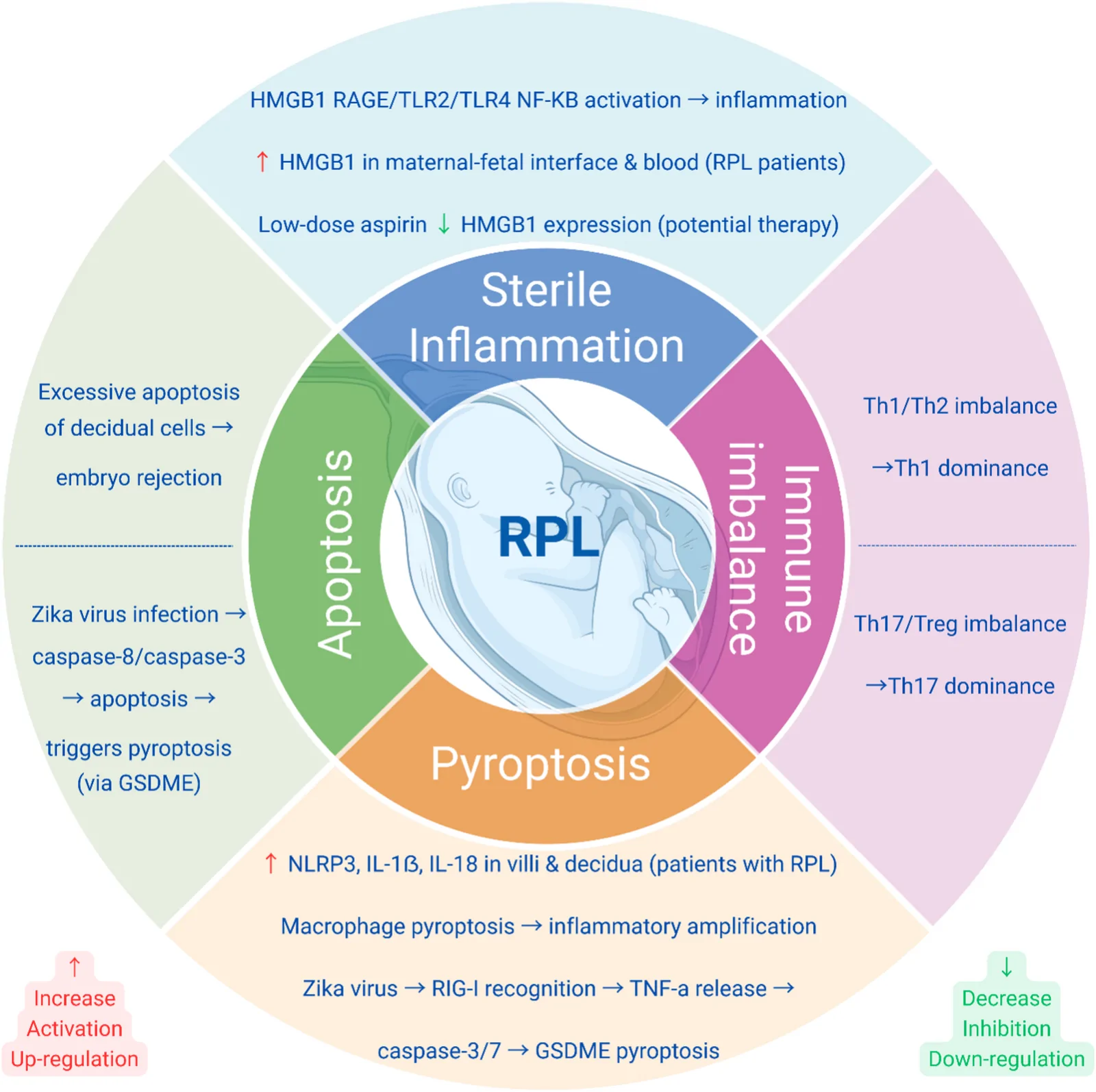
The complex interactions between apoptosis, pyroptosis, and immune dysregulation in recurrent pregnancy loss (RPL). This figure provides a visual overview of the cellular and molecular mechanisms underlying the interplay among sterile inflammation, immune dysregulation, and cell death pathways in the pathogenesis of RPL. Sterile inflammation (blue portion): RPL patients have elevated maternal-fetal interface and circulating levels of high-mobility group box 1 (HMGB1), which activate the receptor for advanced glycation end products (RAGE) and the TLR2/TLR4-NF-κB signaling pathway, ultimately leading to activation of inflammatory pathways. Low-dose aspirin has been shown to suppress HMGB1 expression, suggesting a potential therapeutic approach. Immune dysregulation (purple portion): A Th1/Th2 and Th17/Treg imbalance can lead to greater Th1 and Th17 dominance, which shifts the uterine environment toward pro-inflammatory states that threaten embryo implantation and maintenance. Pyroptosis (orange portion): Increased expression of components of the NLRP3 inflammasome (NLRP3, IL-1β, IL-18) in villi and decidua leads to pyroptosis of macrophages and promotes inflammation. Infection with the Zika virus has been shown to promote pyroptosis via RIG-I recognition of the virus, leading to TNF-α release that fosters macrophage pyroptosis, as well as caspase-3/7 activation, which can then lead to GSDME cleavage. Apoptosis (green portion): Excessive apoptotic decidual cell death contributes to fetal rejection. Viral infections, including those by *Zika virus*, lead to activation of the caspase-8/caspase-3 pathways which drive apoptosis, but can also promote pyroptosis through caspase cleavage of GSDME. Taken together, this figure illustrates overlapping pathways of immune dysregulation that intersects with two death pathways, apoptosis and pyroptosis, due to the effects of inflammasome-activation, that develop sterile inflammation, contributing to fetal rejection

The presence of increased NLRP3 and IL-1β expression in the decidua, together with raised IL-1β and IL-18 blood levels in RPL patients, indicates that macrophage pyroptosis is an important factor in the inflammation that occurs at the interface between the mother and daughter. In a study by Ying Zhao et al., the Th1/Th2 and Th17/Treg balance in the murine spontaneous abortion model skewed toward Th1 and Th17 dominance, respectively, indicating a disrupted immune microenvironment in SA mice. Additionally, the decidua and placenta showed elevated levels of expression of NLRP3, AIM2, caspase-1, ASC, and IL-1β, confirming the existence of an activated pyroptosis and inflammasome at the maternal-fetal interface [117]. Although increasing evidence supports the involvement of inflammasome-associated cell death pathways in endometriosis, several limitations remain. Most available studies are based on in vitro experiments or animal models, whereas mechanistic evidence from human tissues is still limited. Moreover, the relative contribution of apoptosis, pyroptosis, and necroptosis during different stages of disease progression remains unclear. Future studies integrating molecular analyses with clinical data are needed to clarify their temporal and causal relationships. Involvement of different forms of PCD in the pathogenesis of endometriosis are listed in Table 2 and illustrated in Fig. 8.

### Premature ovarian insufficiency (POI)

Ovarian function declines before a woman reaches menopause, a condition known as POI [150, 151]. The primary symptom is a decrease in estrogen levels together with menstruation not occurring for a minimum of four months and FSH values of 25 IU/L or higher on two separate tests spaced four weeks apart [152]. Autoimmune illness, hereditary factors, chemotherapy-induced iatrogenic damage, and enzyme abnormalities are confirmed causes, however the specific etiology is still not fully understood [153].

**Fig. 9:**
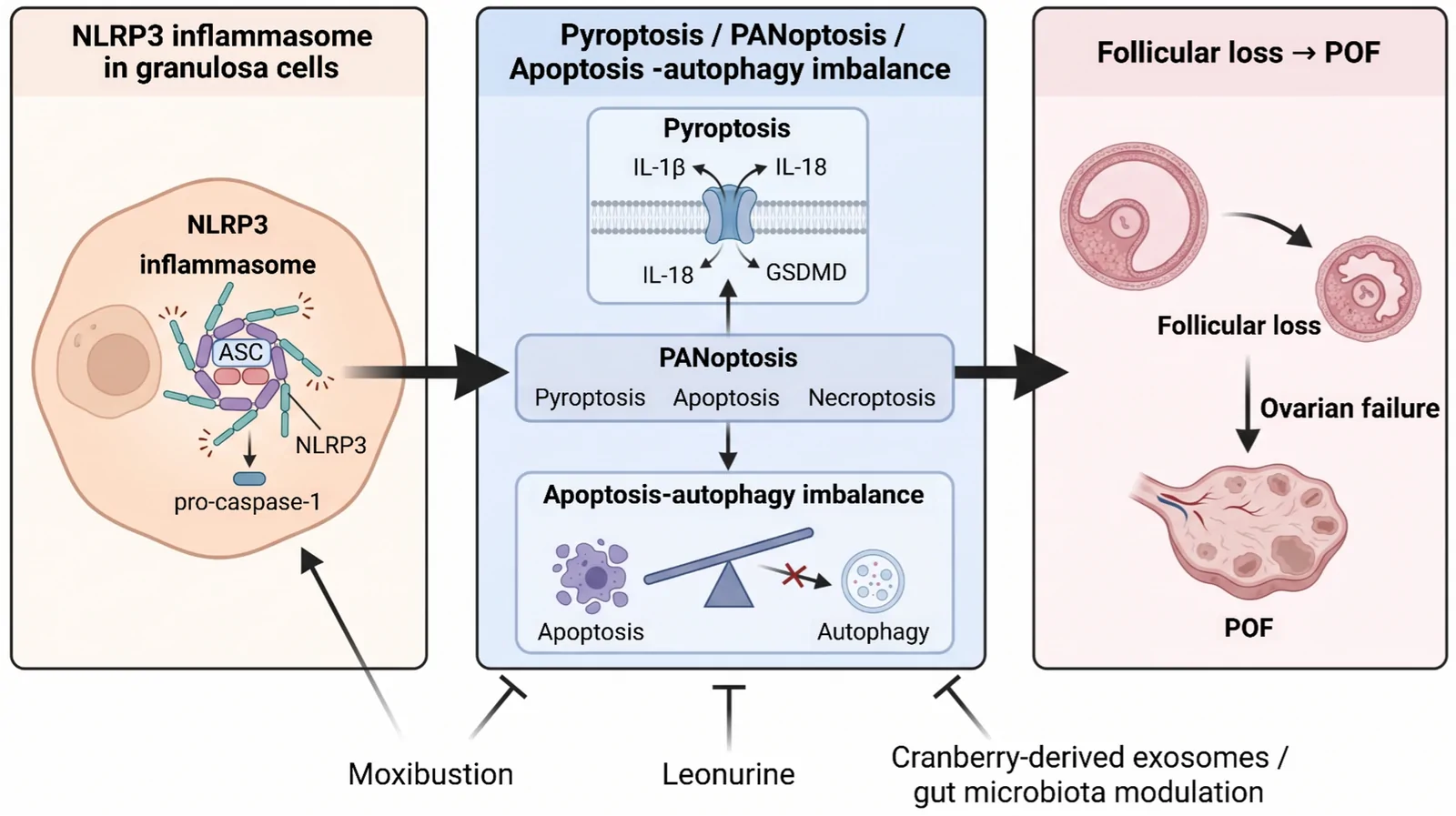
Schematic representation of the roles of pyroptosis, apoptosis/autophagy imbalance, and PANoptosis in premature ovarian insufficiency (POI). In this scheme, we illustrate the interconnections and interactions among PCD pathways to explain how they, together, may lead to POI. Pyroptosis is a vital cell death mechanism, mediated by NLRP3 inflammasome activation, in granulosa cells of patients with POI. Pyroptosis leads to follicular loss and is associated with ovarian failure, as was shown by elevated expression of IL-1β, IL-18, and GSDMD. It is noteworthy that therapeutic interventions such as moxibustion and treatment with leonurine in POI studies have been indicated, in part, to induce POI improvements by suppressing PCD, inflammation, and elevated levels of IL-1β, IL-18, and GSDMD through targeting and/or inhibition of the NLRP3/GSDMD pathways. The role of the apoptosis-autophagy imbalance pathway showed that if NLRP3 was suppressed, less apoptosis and more autophagy could prolong ovarian function. Hence, increased NLRP3 expression is associated with ovarian aging. PANoptosis has recently been shown to occur in granulosa cells of POI models. PANoptosis is considered a new form of integrated PCD featuring aspects of pyroptosis, apoptosis, and necroptosis (PAN). While limited evidence is available, cranberry-derived exosomes were reported. In this paper, POI was attenuated in response to gut microbiota modulation and inhibited PANoptosis. Together, the figure summarizes NLRP3-mediated inflammasome activity and related PCD pathways in regulating the initiation and progression of POI

Evidence suggests that persistent inflammation may cause follicular loss and, ultimately POI. Women with polycystic ovary syndrome have increased NLRP3 inflammasome expression in their ovarian GCs, and inhibiting this gene’s expression in mice improves their fertility [152]. Ovarian function is tightly associated with the NLRP3 inflammasome, as NLRP3 expression in mice ovaries increases with age [154]. A substantial association between POI and NLRP3-mediated pyroptosis has been shown in studies. The binding of NLRP3 to procaspase-1 and subsequent activation of caspase-1 can be triggered by both internal and external stimuli. After that, pyroptosis is triggered when cleaved caspase-1 cleaves GSDMD. Afterwards, inflammation and impaired ovarian function can be caused by activated caspase-1, which enhances the expression of IL-18 and IL-1β [155, 156]. It has been demonstrated that pyroptosis inhibition reduces POI symptoms. Research has shown that moxibustion (Mox) reduced IL-1β, IL-18, and GSDMD, which are important factors in pyroptosis, and so improved cyclophosphamide-induced POI in rats. Research revealed that Mox reduced POI by means of NLRP3-mediated pyroptosis [156]. Consistent with these findings, Yan-Nan Chi et al. showed that leonurine hydrochloride, when administered intraperitoneally, reduced cyclophosphamide-induced POI in mice by inhibiting pyroptosis mediated by NLRP3/GSDMD [118]. It has been indicated that NLRP3 suppression attenuates ovarian aging and prolongs fertility in female mice. The investigation demonstrated that NLRP3 inflammasome ablation in female mice improved metabolic and hormonal characteristics related to female reproductive aging, enhanced autophagy, and reduced apoptosis [157]. One recent study suggested that cranberry-derived exosomes ameliorated experimental POI by suppressing molecular signatures consistent with PANoptosis in ovarian granulosa cells. Nevertheless, additional mechanistic studies are required to verify canonical PANoptosome formation and confirm bona fide PANoptosis in this setting [119]. A clinical study involving 30 women with POI and 30 healthy controls demonstrated increased Th17 cell frequency, elevated serum levels of IL-17, IL-21, and IL-23, and upregulated miR-326 expression in patients with POI, further supporting the contribution of immune dysregulation and chronic inflammation to POI pathogenesis [158]. Evidence supporting the role of inflammasome-mediated cell death in POI is still emerging. Most available data originate from chemotherapy-induced animal models, which may not fully represent the heterogeneous etiologies of human POI. Consequently, the clinical relevance of these findings requires further validation in patient-derived samples. Involvement of different forms of PCD in the pathogenesis of POI are listed in Table 2 and illustrated in Fig. 9.

## Therapeutic implications of inflammasome-associated cell death pathways

### Inflammasome-targeted therapies

One promising strategy to reduce intrauterine inflammation and potentially improve in vitro fertilization (IVF) outcomes is direct inhibition of the NLRP3 inflammasome. The use of MCC950, a small-molecule inhibitor that specifically blocks the activity of the NLRP3 inflammasome while maintaining upstream priming pathways, is a crucial tactic [159]. MCC950 is a promising option for fertility treatment because preclinical studies have shown that it efficiently reduces oxidative damage in inflammatory conditions and inhibits the release of IL-1β [160]. In an experimental study by Thomas van der Heijden et al., apoE-/- mice were treated with MCC950 for 4 weeks to evaluate its effects on atherosclerosis. They showed that MCC950 treatment significantly reduced the development of atherosclerotic lesions and lowered the number of macrophages in the plaque [161]. In another study by Jianhang Jiao and colleagues, it was reported that MCC950 alleviated inflammation and promoted functional recovery in an acute mouse model of spinal cord injury by partly inhibiting NLRP3 inflammasome complex assembly, such as NLRP3-ASC and NLRP3-Caspase-1, as well as by decreasing the release of inflammatory cytokines TNF-α, IL-1β, and IL-18 [162]. Since the NACHT domain binding site for MCC950 is primarily hydrophobic, this may lead to off-target interactions with other proteins containing hydrophobic binding domains and broader immune disruption. Moreover, a humanized monoclonal antibody, IC100 (IgG4), has been developed to target the ASC component of the inflammasome. Desu et al. treated the EAE mouse model of MS with different doses of IC100. They found that treatment with IC100 decreased the recruitment of CD4^+^, CD8^+^ T cells and CD11b^+^MHCII^+^ cells into the CNS [163]. They concluded that targeting ASC with a monoclonal antibody can significantly alter innate and adaptive immune responses in the MOG-induced mouse model, and results in improved clinical outcomes. It should be noted that results from animal models cannot be completely extrapolated and reflect human disease. Further translational studies and clinical trials are required to determine whether these therapeutic strategies can be applied to humans.

Another potential therapeutic strategy involves IL-1β inhibitors, such as neutralizing antibodies and immunizations. The NLRP3 inflammasome induces inflammation by triggering IL-1β. In IVF patients, IL-1β inhibitors may enhance uterine receptivity and reduce chronic inflammation, increasing the chance of a successful implantation. Despite being mostly studied in autoimmune diseases, these inhibitors may improve IVF outcomes [164]. Moreover, recent evidence has highlighted a potential contribution of inflammasome signaling to placental inflammatory disorders. In chronic histiocytic intervillositis (CHI), transcriptomic analyses demonstrated increased activation of the NLRP3-PYCARD inflammasome pathway accompanied by enhanced IL-1β processing within placental tissues. Furthermore, clinical observations in patients with recurrent CHI suggested that anti-inflammatory treatment with anakinra and colchicine was associated with improved pregnancy outcomes and reduced recurrence of intervillositis. These findings support the concept that dysregulated inflammasome activation may contribute to placental inflammation and adverse pregnancy outcomes, while pharmacological modulation of this pathway may represent a promising therapeutic strategy [165].

Because PANoptosis has been implicated in multiple inflammatory conditions across different organ systems, it has emerged as a potential therapeutic target for reproductive disorders. Research on PANoptosis remains in its early stages, and there are still a few challenges, for instance, identifying additional potential sensors and triggers. In addition, the PANoptosome consists of many sensors with functional redundancies, indicating that inflammasomes are an essential element of the PANoptosome. Endometriosis, POI, premature labor, PCOS, RPL, and PE are among the reproductive abnormalities that include the inflammasome in their pathophysiology. The latest findings suggest that a possible approach to treating reproductive diseases could involve targeting the inflammasome and cell death signaling pathways. In this regard, mir-520c-3p has been demonstrated to suppress the NLRP3 inflammasome in PE [166]. Additionally, a decrease of 35.7% in preterm birth and a decrease of 26.7% in neonatal mortality were seen after administering MCC950, an NLRP3 blocker [167]. Treatment of placental diseases with the NLRP3 inhibitor Glibenclamide has similar effects, reducing inflammasome activity in human trophoblast cells [168]. Despite considerable progress in this field, there are still some challenges regarding the safety of NLRP3 inhibitor administration. The consequences of NLRP3 blockade are controversial. NLRP3 is closely associated with key cellular processes including metabolism and autophagy, highlighting the importance of carefully designed and selective inhibitor administration. An essential question that must be addressed is whether complete suppression of NLRP3 is the optimal therapeutic strategy, or whether a context-based modulation of its activity results in better therapeutic outcomes. Chronic inhibition of NLRP3 may disrupt host immune defense and increase the risk of infections. Besides, the potential inflammasome crosstalk must be considered. The inhibition of NLRP3 may be compensated by other pathways such as AIM2, NLRC4, and cGAS, resulting in altered immune response [169].

### Modulation of pyroptosis and inflammatory signaling

NLRP1 and AIM2 inflammasomes are also promising targets for developing therapeutic strategies to treat reproductive disorders. As an example, omega-3 fatty acids have been found to reduce inflammation and infection-related preterm birth by suppressing the activation of NLRP1 and AIM2 inflammasomes and inhibiting the release of cathepsin S from lysosomes into the cytosol [170]. The NLRP3 inflammasome appears to play an important physiological role during early pregnancy and embryo implantation. Experimental studies in mice have demonstrated increased expression of NLRP3, caspase-1, GSDMD, IL-1β, and IL-18 at implantation sites during the peri-implantation period. Pharmacological inhibition of NLRP3 using MCC950 altered inflammasome activity, reduced GSDMD expression, and was associated with a decreased number of implantation sites. These findings suggest that inflammasome activation and inflammasome-dependent pyroptosis are involved not only in pathological reproductive conditions but also in normal implantation and decidualization processes, emphasizing the importance of maintaining a balanced inflammatory response during early pregnancy [171]. Emerging evidence suggests that inflammasome-mediated pyroptosis may contribute to implantation failure and early pregnancy loss through disruption of maternal-fetal immune tolerance. Mechanistically, activation of the NLRP3/ASC/Caspase-1 signaling axis promotes the release of proinflammatory cytokines including IL-1β and IL-18, which may interfere with endometrial receptivity and embryo implantation. Nevertheless, current evidence remains largely indirect, and direct demonstration of pyroptosis in IVF/ICSI-associated early pregnancy loss is still lacking [172]. Recent studies indicate that pyroptosis may play a physiological role during embryo implantation and decidualization. Controlled activation of gasdermin D-mediated pyroptosis in endometrial epithelial cells promotes IL-18 release, which contributes to downstream signaling pathways involved in successful implantation and decidual transformation. However, excessive activation of pyroptotic pathways has been associated with implantation failure and adverse pregnancy outcomes, suggesting that a finely regulated inflammatory balance is essential for reproductive success [173]. Beyond targeting inflammasome-related pathways, immunomodulatory therapies such as intravenous immunoglobulin (IVIG) have also been explored for recurrent pregnancy loss. IVIG is thought to restore immune homeostasis by modulating both innate and adaptive immune responses, highlighting the importance of immune regulation as a complementary therapeutic strategy in reproductive disorders [174]. Taken as a whole, these results from reproductive medicine show how important PANoptosis and the inflammasome are, and they suggest that reproductive diseases can be effectively treated by therapeutically targeting the inflammasome. Based on the available research, prospective therapeutic intervention targets in this sector could be NLRP1-3, AIM2, RIPK1, ZBP1, ASC, caspase-1, and IL-1β. Although direct therapeutic targeting of PANoptosis has not yet been explored in reproductive diseases, regulators such as RIPK1, RIPK3, CASP8, ZBP1, and ASC may represent promising future therapeutic targets. Further studies are needed for developing selective and well-tolerated inhibitory treatments. For example, pyrine domain inhibitors represent a promising option, but their long-term effects need to be carefully investigated. Combination therapies that modulate inflammasome activities may offer safer and more effective therapeutic options.

## Conclusion and future direction

Inflammasomes have emerged as critical regulators of inflammation and programmed cell death within the female reproductive system. Accumulating evidence demonstrates that dysregulation of apoptosis, pyroptosis, and necroptosis contributes to the development and progression of several reproductive disorders, including endometriosis, PCOS, PE, RPL, POI, and implantation-related complications. Among inflammasome complexes, NLRP3 has received the greatest attention because of its ability to promote IL-1β and IL-18 maturation and to drive inflammatory responses that disrupt reproductive tissue homeostasis. Recent advances have revealed extensive crosstalk among different regulated cell death pathways, leading to the concept of PANoptosis as an integrated inflammatory cell death program. Although several reproductive disorders exhibit concurrent activation of apoptosis-, pyroptosis-, and necroptosis-associated signaling pathways, direct evidence demonstrating canonical PANoptosome formation and PANoptosis activation in reproductive tissues remains limited. Therefore, PANoptosis should currently be regarded as a promising and emerging mechanistic framework rather than a fully established pathogenic pathway in reproductive diseases. Therapeutic modulation of inflammasome-associated cell death pathways represents a promising avenue for future intervention. Experimental and preclinical studies have shown encouraging results with NLRP3 inhibitors, IL-1β-targeted therapies, and agents capable of modulating pyroptotic and inflammatory signaling. However, because inflammasome activation also participates in normal physiological processes such as implantation, decidualization, and placental development, future therapeutic strategies should focus on restoring immune and inflammatory balance rather than completely suppressing these pathways. Overall, a better understanding of the molecular interactions among inflammasomes, regulated cell death pathways, and reproductive physiology may facilitate the development of novel biomarkers and targeted therapies for reproductive disorders. Future studies integrating molecular, cellular, and clinical approaches are needed to define the precise contribution of PANoptosis-related mechanisms and to translate these findings into improved reproductive health outcomes.

## Data Availability

No datasets were generated or analysed during the current study.
